# Biophysical and Molecular mechanisms that control active wetting and tissue fluidification in epithelial tissues

**DOI:** 10.21203/rs.3.rs-6008502/v1

**Published:** 2025-03-03

**Authors:** Stefano Marchesi, Chiara Guidolin, Andrew E. Massey, Gregoire Lemahieu, Zeno Lavagnino, Galina V. Beznoussenko, Alexandre A. Mironov, Brenda Green, Elisa Allievi, Emanuele Martini, Serena Magni, Andrea Ghisleni, Caterina Lomazzi, Andrea Francesco Benvenuto, Andreas Schertel, Dario Parazzoli, Paolo Maiuri, Sara Sigismund, Nils Gauthier, Ada E. Cavalcanti-Adam, Alexander X. Cartagena-Rivera, Fabio Giavazzi, Giorgio Scita, Andrea Disanza

**Affiliations:** 1 IFOM ETS, the AIRC Institute of Molecular Oncology, Milan, Italy; Department of Oncology and Haemato-Oncology, University of Milan, Milan, Italy.; 2 Department of Medical Biotechnology and Translational Medicine, University of Milan, Segrate, Italy.; 3 Section on Mechanobiology, National Institute of Biomedical Imaging and Bioengineering, National Institutes of Health, Bethesda, MD, USA.; 4 Department of Cellular Biophysics, Max Planck Institute for Medical Research, Heidelberg, Germany.; 5 Department of Oncology and Haemato-Oncology, University of Milan, Milan, Italy.; 6 Present address: Fondazione Human Technopole, Milan, Italy.; 7 European Institute of Oncology (IEO) IRCCS, Milan, Italy.; 8 Carl Zeiss Microscopy GmbH, Oberkochen, Germany.; 9 Present address: Dipartimento di Medicina Molecolare e Biotecnologie Mediche, Università degli Studi di Napoli Federico II, Naples, Italy.; 10 Department of Cellular Biophysics, Max Planck Institute for Medical Research, Heidelberg, Germany; Cellular Biomechanics, University of Bayreuth, Bayreuth, Germany.; 11 Lead contacts

## Abstract

Tissue-level phase transitions are emerging as a crucial mechanism in tumour development and metastasis. This study aims to identify molecular determinants and physical conditions that control active wetting and solid-to-fluid transition of epithelial tissues. We focused on IRSp53, a protein linking plasma membranes to the cytoskeleton. Depleting IRSp53, in MCF10 DCIS.com cells, disrupts coordinated collective movement by promoting local fluctuations in cell velocity resulting in increased tissue fluidity. In dense monolayers, IRSp53 ablation allows cells to escape the physical constraint imposed by cell crowding resulting in a delayed transition toward a jammed state. In 3D spheroids, IRSp53 loss fosters active wetting of a rigid substrate, shifting spheroid behaviour to a more fluid-like state. Biophysical modelling of the spreading cells as an active polar fluid indicates that IRSp53 depletion reduces bulk viscosity and contractility in spheroids. This effect is the result of reduced supracellular tension and disrupted organization of cell-cell junctions, which lead to decreased intercellular friction and enhanced local cell rearrangements. Molecularly, IRSp53 physically and functionally interacts with the junctional protein Afadin in the regulation of tissue tensile state and active wetting in tumour spheroids. These findings identify IRSp53 and Afadin as key regulators of tissue viscosity in breast cancer tumoroid undergoing solid-to-fluid transition linked to tumour progression. They further provide the molecular basis to causally relate subcellular and cell scale processes to tissue-levels dynamics.

## Introduction

Alteration of the mechanical properties of single cells and tissues, determining their architecture, composition and function, are associated with several diseases, most notably cancer^1^. Cancer tissues are intrinsically linked to the mechanical properties of their cellular subcomponents^2,3^. Tensional, compressive, adhesive, elastic and viscous properties are mostly regulated by reorganization of the actomyosin cytoskeleton, which transmits forces at tissue level by establishing cell-cell and cell-extracellular matrix (ECM) adhesions.

At tissue-level, solid-to-liquid-like and active wetting phase transitions (PT) are emerging as key determinants of cell behaviour and fate^4–10^. For example, normal epithelial glandular organs often adopt a solid-like state at a critical density, which depends on several biophysical parameters, such as intercellular adhesion, cortical tension, single-cell motility and cell shape variance^10^. This PT ensures the correct development of barrier and elastic properties in normal epithelial tissues and serves as a tumour-suppressive mechanism by preventing the emergence of aberrant, fluid-like clones^5,11,12^. However, disruptions in cell-cell and cell-ECM adhesions, alterations of traction forces, and changes in cell shape can drive a transition toward a more fluid-like state, which has been linked to aggressive forms of cancer^5,11^. These factors are crucial to the transition from a jammed (solid-like) to an unjammed (fluid-like) state, a process recently identified as a driver of the transition from indolent in situ ductal carcinoma (DCIS) to invasive malignancy during breast cancer^5,11,12^.

A process that may recapitulate some aspects of the progression of DCIS to invasive and spreading carcinoma is the transition between three-dimensional spheroidal aggregates and two-dimensional epithelial monolayers, which can be understood as an active wetting transition^5,6^. Active wetting of epithelial tissue cannot be explained exclusively in terms of the physics of passive fluids^9^. Instead, active epithelial tissue wetting dynamics has been captured by a framework based on an active polar fluid model of tissue spreading^9^, whereby the transition results from the competition between traction forces and contractile intercellular stresses.

Despite the advances in the physical understanding of phase transitions, the molecular players the cells use to control these processes are poorly defined.

To gain insights into this direction, we employed 3D spheroid wetting as a model system, where spheroidal cell aggregates transition into spreading monolayers that “wet” the substrate^6,9,13^. Analyzing the dynamics of this process is expected to shed lights into how changes in cellular viscosity, supracellular tension, and junctional integrity contribute to tumor progression and metastasis. By combining this approach with advanced imaging, biophysical measurements, and modeling, we aimed to uncover the molecular and physical conditions that drive the transformation of normal, solid-like tissues into cancerous, fluid-like states.

We hypothesized that proteins that link the plasma membranes with the underlying cytoskeleton in epithelial tissues may be key regulators in the generation and transmission of the physical forces between adjacent cells that control supracellular cohesion, guide collective motion, and are at the base of the jammed-to-unjammed and wetting transitions.

The family of proteins that contains the membrane-bending and -binding I-BAR domain has all the key structural features to perform this function^14^. Among these proteins, IRSp53, the founding member of this family, stands out as it regulates the dynamic interplay between the plasma membrane (PM) and the actin cytoskeleton by sensing and promoting membrane curvature through its I-BAR domain and interacting with various actin regulatory proteins^14–16^. Through these activities, IRSp53 promotes directional migration by inducing filopodia and lamellipodia^16–18^. Of note, similar structures were also reported to shape the architecture of cell-cell adhesion in mature epithelia^19,20^. Accordingly, IRSp53 has been shown to play a role in cell–cell adhesion, where it is enriched^21–23^, and to regulate the internalization and trafficking of cell–ECM adhesive receptor β1-integrin^24^. In addition, it is required for the polarized architectural organization and morphogenesis of epithelial tissues^21^. In these processes, IRSp53 provides a membrane curvature-sensing/deforming platform for the assembly of multi-protein complexes that control the trafficking of apical determinants and the integrity and shape of the luminal plasma membrane^21^. The disruption or loss of these mechanisms is characteristic of collective breast-epithelial tumour locomotion, making IRSp53 a prime candidate to be characterised in this context.

We focused our investigations on IRSp53’s function within the framework of collective cell movement, utilizing both 2D and 3D assays of MCF10 DCIS.com cells, used as models of early breast cancer development^25^. Our findings indicate that the depletion of IRSp53 compromises the ability of cells to move collectively in a coordinated fashion during wound healing. In monolayers that undergo a rigidity transition due to increased cell density, IRSp53 ablation allows cells to escape the physical constraint imposed by cell crowding resulting in delayed jamming transition. Using normal and tumorigenic 3D spheroids that undergo a wetting transition^6,9,13^, we showed that loss of IRSp53 robustly accelerates wetting rates. Biophysical modelling of this process, based on a modified active polar fluid model^9^ indicated that IRSp53 controls spheroid viscosity rather than cell-substrate interaction, which we verified experimentally in multiple ways. Reduction of viscosity in IRSp53 depleted cells is the result of reduced supracellular tension and perturbed mechanical and architectural organization of cell-cell junctions, which lead to decreased intercellular friction and enhanced local cell rearrangements (tissue fluidity).

At the molecular level, we investigated the IRSp53 junctional interactome^21^ and we uncovered a novel functional interaction between IRSp53 and the junctional protein Afadin (AFD). Afadin, which is essential for development^26–28^, has been previously shown to influence the mechanical properties of epithelial cells and regulate epithelial polarity, similar to IRSp53^29,30^. We found that Afadin forms a complex with IRSp53, and its depletion mimics the effects of IRSp53 loss in 3D spheroid wetting assays in controlling supracellular tensile state.

Together, IRSp53 and Afadin emerge as pivotal regulators of junctional organization and the viscoelastic properties of cell collectives. By controlling solid-to-fluid transitions, these molecules play a critical role in tumor progression, bridging fundamental cellular-scale processes with tissue-scale dynamics.

## Results

### IRSp53 removal affects DCIS collective motion

IRSp53 regulates the dynamic interplay between the PM and the actin cytoskeleton during directional migration and invasion, has a role in cell–cell and cell–ECM adhesions, and is required for the polarized architectural organization and morphogenesis of epithelial tissues^14–18,31^. Perturbation or loss of these mechanisms is associated with invasive breast cancer that acquires collective locomotory behavior^32^.

**Figure 1.**
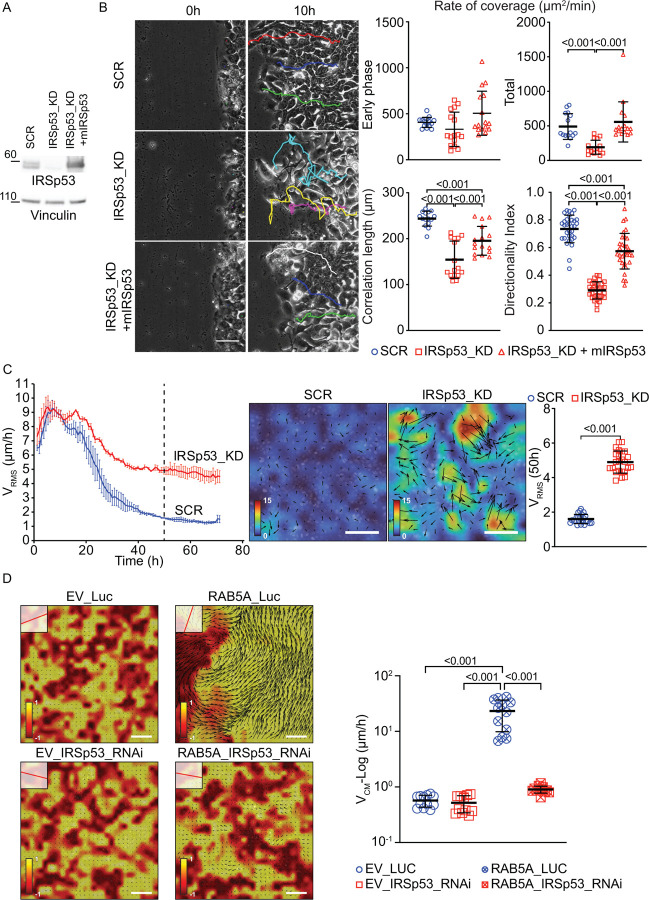
IRSp53 removal affects DCIS collective motion. **A.** Immunoblots of doxycyclinetreated MCF10 DCIS.COM control (SCR), IRSp53 silenced not expressing (IRSp53_KD) or expressing murine IRSp53 (IRSp53_KD + mIRSp53) with the indicated antibodies. **B.** Left images: scratched wound migration of doxycycline-treated MCF10 DCIS.COM SCR, IRSp53_KD or IRSp53_KD + mIRSp53 monolayers (Supplementary Movie S1). Representative still images at the indicated time points are shown. Scale bars, 100μm. Right, cell motility analysis. Upper graphs, the rate of coverage was quantified by measuring the percentage of area covered by cells over time (Early phase represents the 2 hours of active migration). Lower left graph, correlation length was obtained by PIV analysis. Data are mean ± SD (at least 15 fields of views were analysed from 3 independent experiments). Lower right graph, the directionality index was obtained by manually tracking single cells at the migrating front (example trajectories are shown) by ImageJ. Data are mean ± SD (n=32 cells from 3 independent experiments). **C.** Left, root mean square velocity $\left({\text{V}}_{\text{RMS}}\right)$ over time, obtained from PIV analysis, for doxycycline-treated SCR-H2B-GFP and IRSp53_KD-H2B-mCherry MCF10 DCIS.COM cells seeded at jamming density and monitored by time-lapse microscopy (Supplementary Movie S4). Data are mean ± SD (> 20 fields from 3 independent experiments). The vertical dashed line indicates the time point (t=50 h) corresponding to the snapshots shown beside. Centre: representative snapshots of the velocity field. The colormap reflects the magnitude of the local velocity (μm/h) as indicated by the colour bar. Scale bar 100μm. Right: ${\text{V}}_{\text{RMS}}$ at t=50h. Data are mean ± SD(> 20 fields from 3 independent experiments). **D.** Left: Representative snapshots of the velocity field (t=20h) obtained from PIV analysis of doxycycline-treated empty vector (EV) and RAB5A MCF10 DCIS.COM cells, silenced with oligos for Luciferase (EV_Luc, RAB5_Luc) or human IRSp53 (EV_IRSp53_RNAi, RAB5_IRSp53_RNAi) seeded at jamming density and monitored by time-lapse microscopy (Supplementary Movie S5). The colour-map reflects the alignment with respect to the mean velocity v_CM, quantified by the parameter $\text{a}(\text{x})=v(\text{x})\cdot v\_\text{CM}/\|v(\text{x})\|\left\|v\_\text{CM}\right\|$. A value a=1 (a=−1) indicates that the local velocity is parallel (antiparallel) to the mean direction of migration, indicated by the red line in the upper left square of each snapshot. Scale bar 200μm. Right: collective motion velocity V_CM shown in Log scale (n=12 fields from 2 independent experiments). IRSp53 and RAB5A expression was verified by qRT-PCR (Supplementary Fig. S1C). Statistical analysis for each experiment is included in the Methods section. P values are indicated in each graph.

To investigate the role of IRSp53 in this context, we developed shRNA-expressing MCF10 DCIS.com cells to knock down IRSp53 in an inducible fashion (Fig. 1A). MCF10 DCIS.com cells are an oncogenic T24 H-RAS expressing human mammary epithelial cells, which can generate ductal mammary carcinoma when injected into immunocompromised mice that eventually progress to become invasive carcinoma^25^. These tumors recapitulate the natural history and evolution of human DCIS lesions and display a plastic EMT state, retaining epithelial markers and features, such as the expression of junctional E-cadherin, and the ability to form rigid and jammed monolayers, but also some mesenchymal traits^12^. Using this model, we first examined the impact of IRSp53 on directional collective migration during wound repair in confluent epithelial monolayers. IRSp53 depletion resulted in marginal effects on the initial rate of wound closure, compared to control cells (SCR) (Fig. 1B, Rate of coverage – Early Phase, Supplementary Movie S1), but significantly reduced correlation length, and directionality in single-cell trajectories, which eventually caused a delay in closing the wound at later time points (Fig. 1B, Supplementary Movie S1). However, both wound closure as well as long-range coordinated and directional motion could be restored when murine IRSp53, resistant to the shRNA, was re-expressed in IRSp53 silenced cells (Fig. 1A–B, Supplementary Movie S1). Importantly, IRSp53 loss did not affect the migration of individual cells in one-dimensional (1D) linear motility assays (Supplementary Fig. S1A, Supplementary Movie S2), indicating that IRSp53 impacts emergent properties of the cell collective.

The loss of coordination was not limited to the leading edge of migrating cells but also occurred far from the front within the monolayer (Supplementary Movie S3). This suggested that IRSp53 is crucial to enable long-range coordination of motion in epithelial monolayers. Like the parental normal mammary MCF10A cells, MCF10.DCIS.com cells form tightly packed and dense epithelial monolayers. Upon proliferation, the motion of each individual cell progressively ceases constrained by the crowding due to its neighbors. This phenomenon can be described as a fluid-to-solid (jamming) phase transition, where the cell monolayer undergoes a shift from a more fluid-like, mobile state to a solid-like, stationary, and mechanically rigid state. In this jammed state, cell motility is significantly reduced as crowding leads to physical constraints on movement^10^.

Notably, the silencing of IRSp53 impeded the transition to a jammed state in MCF10 DCIS.com cells. Mature IRSp53 knockdown (KD) monolayer retained a fluid-like motility, characterized by large velocity fluctuations determined by measuring the root mean square velocity $\left({\text{V}}_{\text{RMS}}\right)$ (Fig. 1C, Supplementary Movie S4). Thus, IRSp53 is a critical determinant of the jamming transition in dense cell monolayer. Despite the effect on collective motility, the downregulation of IRSp53 did not affect the rate of cell proliferation deduced from plotting the time evolution of nuclear density (Supplementary Fig. S1B). This suggests that IRSp53 specifically influences physical and mechanical properties of the epithelial monolayer, rather than directly affecting cell division.

Previous research showed that the expression of the endocytic protein RAB5A is sufficient to reawaken cell motility in kinetically arrested monolayers by promoting long-range coordinated motion that results in flocking where multiple cells align their velocity vectors and local fluctuation in cell velocity that effectively fluidizes the system^7,12,33^. Disrupting the ability of a cell to align its locomotion with that of neighboring cells is expected to impair flocking behavior.

To explore the impact of IRSp53 on the emergence of RAB5A-induced flocking motion, we silenced IRSp53 in RAB5A-expressing MCF10.DCIS.com monolayer. As expected, RAB5A reawakened cell motility, promoting the highly coordinated motion of multicellular streams that is quantitatively captured by measuring the mean velocity of the center of mass $\left({\text{V}}_{\text{CM}}\right)$ extracted from the particle image velocimetry (PIV) analysis (Fig. 1D, Supplementary Fig. S1C and Supplementary Movie S5). However, in the absence of IRSp53, flocking locomotion was severely compromised as indicated by a robust and significant reduction in the mean ${\text{V}}_{\text{CM}}$ (Fig. 1D, Supplementary Fig. S1C and Supplementary Movie S5).

Taken together, these findings highlight the critical role of IRSp53 in facilitating long-range coordinated collective motility. Specifically, IRSp53 appears essential for enabling individual cells to align their locomotion with that of their neighbors, a process fundamental for flocking behavior.

### Biophysical modelling reveals that IRSp53 loss decreases tissue viscosity without impinging on cell-substrate adhesion and traction forces.

Next, we investigated what physical processes IRSp53 may regulate to control tissue dynamics and coordinated cell locomotion. We employed normal and tumorigenic 3D spheroids that undergo an active wetting transition^6,9,13^. These 3D spheroids transition into 2D spreading monolayers that “wet” the substrate.

**Figure 2.**
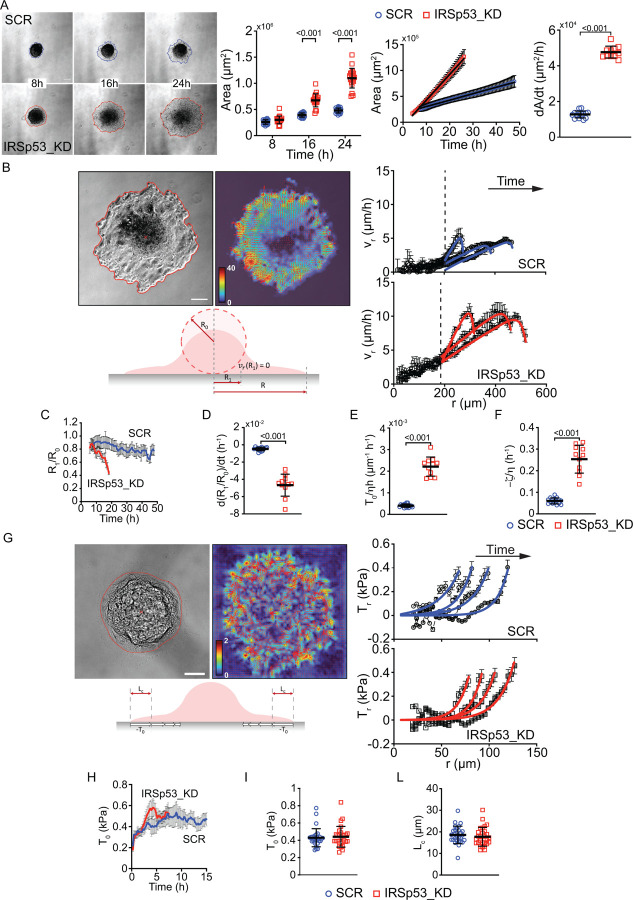
IRSp53 loss decreases tissue viscosity. **A.** Doxycycline-treated MCF10 DCIS.COM control (SCR) or IRSp53 silenced (IRSp53_KD) cells were cultured in ultra-low adhesion condition to form spheroids. Spheroids were then seeded on fibronectin coated 6 well plates and spreading monitored by time-lapse microscopy (Supplementary Movie S6). Representative still images at the indicated time points are shown. Scale bar, 200μm. Left graph, spreading area at the indicated time points was manually quantified using ImageJ. Data are mean ± SD (n=21 SCR, 20 IRSp53_KD spheroids from 3 independent experiments). Right graphs, the spreading area was also quantified with semi-automatic image segmentation with MATLAB to follow its evolution over time. The time evolution of the area for SCR and IRSp53-KD samples shows a significantly faster spreading upon IRSp53 knockdown. The solid black lines represent linear fits to the data, having a slope $\text{dA}/\text{dt}=(1.27\pm 0.19{)}^{*}10^{4}\;\mu {\text{m}}^{2}/\text{h}$ for SCR and $\text{dA}/\text{dt}=(4.77\pm 0.33{)}^{*}10^{4}\;\mu {\text{m}}^{2}/\text{h}$ for KD. The rates dA/dt quantified for single samples are shown in the boxchart. Data are mean ± SD (n=16 SCR, 11 IRSp53_KD spheroids from 3 independent experiments). **B.** PIV. Upper left: representative still images at t=24 h showing the spreading of a (IRSp53-KD) spheroid sample. The image on the left shows the spheroid as seen from the top with the segmentation of the spreading area outlined in red, while the frame on the right shows the corresponding local velocity field obtained from PIV analysis. The colormap represents the magnitude of the local velocity expressed in μm/h. Scale bar 200μm. A side-view schematic illustration of a spreading spheroid is reported below for a visual representation of the main geometrical parameters. The radius ${\text{R}}_{0}$ is the initial spheroid size, calculated as the equivalent radius of its projection at time t=0. The radius R represents the distance of the spreading front from the center of mass and is evaluated as the equivalent radius of the spreading area. From the PIV velocity field, we calculate the azimuthally averaged radial velocity profile, and we monitor its evolution over time. The radius ${\text{R}}_{1}$ represents the size of the stiff core and is the point at which the radial velocity component is expected to go to zero. Right: examples of radial velocity profiles at different times for SCR and IRSp53-KD spheroids. By fitting the model to these profiles, we extract information on tissue viscosity and contractility from the fit parameters. **C.** Time evolution of the radius ${\text{R}}_{1}$ normalized by the initial spheroid size ${\text{R}}_{0}$. While ${\text{R}}_{1}/{\text{R}}_{0}$ remains almost constant for SCR, a fast decrease is observed upon IRSp53 knockdown, mirroring a faster melting of the ‘solid’ core. **D**. The melting rates $d\left({\text{R}}_{1}/{\text{R}}_{0}\right)/dt$ calculated from linear fits of ${\text{R}}_{1}(\text{t})/{\text{R}}_{0}$ for single spheroids highlight the difference between SCR and IRSp53-KD. Data are mean ± SD (n=15 SCR, 11 IRSp53_KD spheroids from 3 independent experiments). **E-F.** The fit of the radial velocity profiles also gives the parameters A and B, allowing for a comparison of the ratio between the traction ${\text{T}}_{0}$ and the viscosity η (A), as well as of the ratio between the contractility ζ and viscosity (B). Data are mean ± SD (n=16 SCR, 11 IRSp53_KD spheroids from 3 independent experiments). **G.** Traction force microscopy. Upper left: representative still image showing the segmentation of a (IRSp53-KD) spheroid on the left and the corresponding traction map on the right. The colormap represents the magnitude of the local traction expressed in kPa. Scale bar 50μm. A side-view schematic illustration of a spreading spheroid is reported below for a visual representation of the main geometrical parameters. We calculate the radial traction profiles ${\text{T}}_{\text{r}}(\text{r})$ at different times and, by fitting the model to these profiles, we quantify the nematic length Lc and the maximum traction $T_{0}$, highlighted in the schematic illustration. Right: examples of radial traction profiles at different times for SCR (top) and IRSp53-KD (bottom) spheroids. Blue (SCR) and red (IRSp53 KD) solid lines are the best fits to the data. **H.** Average evolution of the maximum traction $T_{0}$ over time for SCR (blue line) and IRSp53_KD (red line). **I-L.** Maximum traction $T_{0}$ and average nematic length $L_{c}$ evaluated at the same spreading, when the spreading front has doubled the initial spheroid size, namely R=(2.0±0.2) ${\text{R}}_{0}$. Data are mean ± SD (n=29 SCR, 28 IRSp53_KD spheroids from 3 independent experiments). We remark that no significant difference in ${\text{L}}_{c}$ or ${\text{T}}_{0}$ is observed between SCR and IRSp53_KD. Statistical analysis for each experiment is included in the Methods section. P values are indicated in each graph.

Using time-lapse microscopy, we monitored the wetting dynamics of control (SCR) and IRSp53 KD spheroids on adhesive substrates. MCF10.DCIS.com spheroids, each composed of a predefined number of cells, were generated by seeding doxycycline-treated cells under low-adhesion conditions. Surprisingly, despite the loss of IRSp53 reduced directed, coordinated motion during wound healing (Fig. 1B), it significantly accelerated spheroid spreading under these conditions (Fig. 2A and Supplementary Movie S6). This observation was corroborated using primary murine mammary epithelial cells (MECs) derived from IRSp53-null mice (Supplementary Fig. S1D and Supplementary Movie S7), as well as human HaCat keratinocytes with IRSp53 downregulated via inducible shRNA (Supplementary Fig. S1E and Supplementary Movie S8).

Altogether, these results provide genetic evidence that IRSp53 is crucial for regulating wetting dynamics. They further indicate that IRSp53 plays a general role in controlling this process in both tumorigenic and non-tumorigenic cell assemblies.

Analogous to the behavior of liquid droplets, the propensity of cell aggregates to spread on a substrate depends on the balance between cell cohesion (cell-cell adhesion energy) and the generation of traction forces on the substrate (cell-substrate adhesion energy), which drive the expansion of the spreading sheet^6,9^. Since IRSp53 loss impairs wound closure dynamics (Fig. 1B), a process that shares features with the spreading of a cell sheet in 2D, it is unlikely to affect cell-substrate adhesion and sheet expansion significantly, suggesting, instead, that IRSp53 primarily regulates spheroid cell cohesion under these conditions. We investigated this further below.

In first approximation, both SCR and IRSp53_KD cells expand at a roughly constant rate $\frac{dA}{dt}$. The value however dramatically changes between the two cases, the spreading of SCR spheroids $\left(\frac{dA}{dt}=(1.27\pm 0.19)\cdot 10^{4}\;\mu m^{2}\;h^{-1}\right)$ being significantly lower than IRSp53_KD one $\left(\frac{dA}{dt}=\right.(4.77\pm 0.33)\cdot 10^{4}\;\mu m^{2}\;h^{-1})$(Fig. 2A). The area growth rate depends on the initial spheroid size but a significant difference between SCR and KD is observed over a wide range of spheroid sizes (Supplementary Figure S1I).

To get further insight into the spreading dynamics, we measured the time-resolved cell velocity field via PIV analysis of the phase contrast time-lapse acquisitions. The resulting radial velocity profiles, $v_{r}(r,t)$ display a distinctive non-monotonic shape featuring a peak close to the boundary of the spreading monolayer (Fig. 2B), in qualitative agreement with the predictions of the model introduced earlier^9^.

Briefly, the model describes the cell monolayer as a two-dimensional active polar fluid, characterized in terms of a polarity field p and a velocity field v. In this model, the polarity field follows purely relaxational dynamics, which is assumed to be faster than the spreading dynamics. Under this adiabatic approximation, the equation for p are time-independent and reads

(1) $$L_{c}^{2}\nabla^{2}p_{\alpha }=p_{\alpha },$$

where $L_{c}$ is a nematic length characterizing the persistence of planar polarity, or stated differently, it is a measure of the distance over which cell alignment persists. In the low-Reynolds number limit, the momentum conservation reduces to a force balance condition. Neglecting hydrostatic contributions, force balance requires the traction stress T associated with forces exerted by substrate be equal to the gradient of the monolayer internal tension $\sigma^{s}/h$

(2) $$\frac{1}{h}\partial_{\beta }\sigma_{\alpha \beta }^{s}=T_{\alpha },$$

where h is the monolayer thickness and $\sigma^{s}$ is symmetric part of the stress tensor. The antisymmetric part, consistently with the adiabatic approximation for the polarity field, is assumed to be negligible. The model is complemented with the following simplified constitutive equations

(3) $$\sigma_{\alpha \beta }^{s}=\eta \left(\partial_{\alpha }v_{\beta }+\partial_{\beta }v_{\alpha }\right)-\zeta p_{\alpha }p_{\beta },$$

and

(4) $$T_{\alpha }=-T_{0}p_{\alpha },$$

for the internal stress and the traction stress, respectively. In previous equations, $T_{0}$ represents the maximal traction stress, which quantifies the maximal force per unit area exerted by cells on the substrate; ζ<0 is the active stress coefficient or contractility, which is related to the active contractile forces within the tissue, driven by molecular motors like myosin; and η is the monolayer viscosity, representing its resistance to flow. Remarkably, as shown before^9^, the model can be analytically solved in a circular geometry, assuming purely radial polarization and velocity fields. The circular monolayer (or radius R) is assumed to be fully polarized at the boundaries, leading to the boundary condition $p_{r}(R)=1$ for the radial component of the polarity field, while symmetry considerations impose $p_{r}(0)=0$ at the center. Similarly, the radial component of the velocity field is assumed to vanish at the center $v_{r}(0)=0$, while stress-free boundary conditions $\sigma_{rr}(R)=0$ are imposed on the free edge of the monolayer.

Here, to account for the presence at the center of spreading monolayer of a persistent 3D solid aggregate, we solved the model in an annular domain of external radius R and internal radius $R_{1}$, corresponding to the size of the stiff core. Consistently, at the internal boundary, we assume $v_{r}\left(R_{1}\right)=0$ and $p_{r}(0)=0$, while the conditions at the external boundary are the same as in^9^. More details can be found in Material and Methods section.

We used the analytical solution $v_{r}(r)$ as a fitting model for our velocimetry data (Fig. 2B). The model accurately fits the experimental data. Fitting the solution to the data enables estimating, for each experiment and each time point, $R_{1}$, two combinations of physical parameters, namely $A\equiv T_{0}/\eta h$ and $B\equiv -\frac{\zeta }{\eta }$, and the nematic length $L_{c}$ (Fig. 2E–F and Supplementary Fig. S1F–G) (see Material and Methods section for details).

While $L_{c}$ displays only slight differences between SCR and IRSp53 KD spheroids (Supplementary Fig. S1G, N), the temporal evolution of $R_{1}$ reveals notable disparity. Specifically, the dissolution of the solid aggregate at the center of the spreading monolayer is significantly slower in control spheroids compared to IRSp53 KD ones (Fig. 2C), as mirrored by the reduction in the normalized melting rates $\text{d}\left({\text{R}}_{1}/{\text{R}}_{0}\right)/\text{dt}$ (Fig. 2D and Supplementary Fig. S1O). This striking difference qualitatively accounts for the markedly reduced initial spreading rate observed in control spheroids relative to IRSp53 KD spheroids (Fig. 2A), suggesting that the primary process limiting tissue spreading in control spheroids is the fluidization and dissolution of the spheroid structure. Moreover, both $T_{0}/\eta h$ and $-\frac{\zeta }{\eta }$ are significantly increased in IRSp53 KD aggregates (Fig 2E–F). This result, which is found to be robust across a wide range of spheroid sizes (Fig. S1L–M), indicates that the loss of IRSp53 in spreading spheroids impacts both the ratio between traction forces exerted on the substrate and viscosity, and the ratio between tissue contractility and viscosity. The ratio $\text{B}/\text{A}=-\frac{\zeta h}{T_{0}}$ exhibits a reduction upon IRSp53 knockdown (Fig. S1H), which suggests an impact also on the ratio between tissue contractility and traction, independently of viscosity.

To further investigate these possibilities and dissect the parameters contributing to the parameters A and B, we used traction force microscopy to directly measure the traction stresses exerted by cell aggregates on the substrate during spreading (Fig. 2G and Supplementary Movie S9). We obtained radial traction profiles $T_{r}(r,t)$ featuring a monotonic decay moving from the boundary of the spreading monolayer to the center (Fig. 2G), in excellent agreement with the prediction of the active polar fluid model (see Material and Methods section for details). Fitting the model to the experimental data allowed us to estimate the polar length $L_{c}$ and the maximal traction stress $T_{0}$. Notably, no significant differences were observed in $L_{c}$ or $T_{0}$ between control and IRSp53 KD cells (Fig. 2H–L). Additionally, the $L_{c}$ estimates were consistent with values independently derived from PIV data (Supplementary Fig. S1 G, N). Our results demonstrate that traction stress remains unchanged following IRSp53 knockdown, indicating that the primary effect of IRSp53 depletion is a significant reduction in tissue viscosity and contractility.

We also monitored the size and number of focal adhesions, which have been shown to be directly dependent on the forces exerted to the substrate^34^. The loss of IRSp53 did not alter the number and size of focal adhesions (Supplementary Fig. S2A).

Moreover, IRSp53 was recently reported to be part of an endocytic machinery that is critical for β1integrin endocytosis and is involved in focal adhesion formations^24^. However, the loss of IRSp53 had no impact on the levels of total and active β1-Integrin on the cell surface, implying that IRSp53 is not, or only marginally involved in integrin β1 trafficking in our DCIS cellular system (Supplementary Fig. S2B)

Taken together, these results indicate that IRSp53 controls the rate of spreading of epithelial spheroids by primarily regulating tissue viscosity and contractility.

### Independent experimental validation of the role of IRSp53 in controlling tissue viscosity.

To experimentally validate the role of IRSp53 in the regulation of spheroid viscosity we took different approaches.

**Figure 3.**
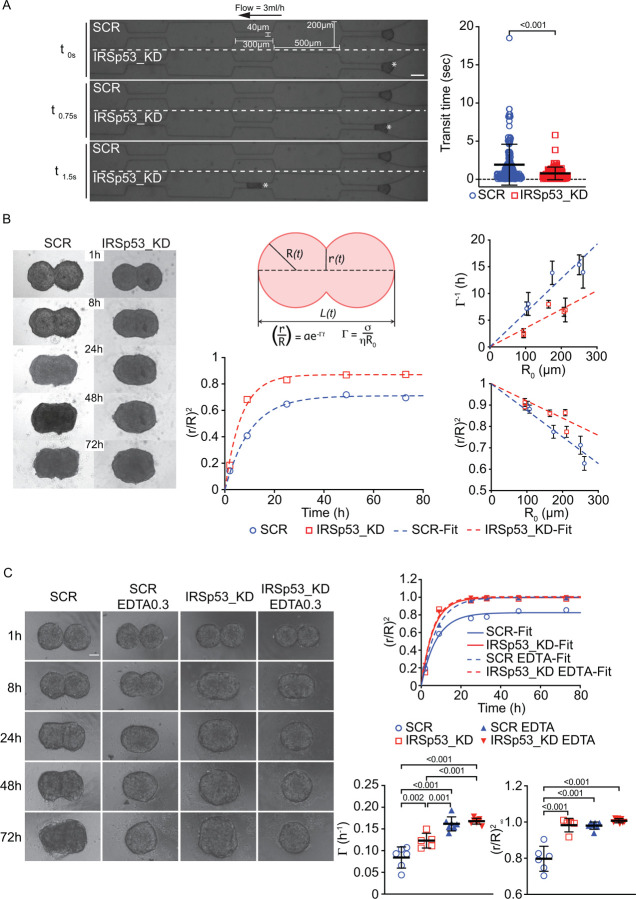
IRSp53 controls tissue viscosity. Validation. **A.** Doxycycline-treated MCF10 DCIS.COM control (SCR) or IRSp53 silenced (IRSp53_KD) cells were cultured in ultra-low adhesion condition to form spheroids. Spheroids were then flowed into a microfluidic device designed for repetitive deformations (B.G. and G.S., manuscript in preparation). Representative still images showing a top-view of MCF10 DCIS.COM spheroids entering the first three constrictions of the deformation device (Supplementary Movie S10). Channel dimensions are shown (height 190μm). Scale bar, 100μm. Right, transit time in the first channel was estimated by manually tracking single spheroids using ImageJ. Data are mean ± SD (n=261 SCR, 248 IRSp53_KD spheroids from 3 independent experiments). **B.** Left, doxycycline-treated MCF10 DCIS.COM control (SCR) or IRSp53 silenced (IRSp53_KD) cells were cultured in ultra-low adhesion condition to form spheroids of different size (500, 2500 and 5000 cells). Pairs of spheroids of the same size were then mixed, and fusion was monitored over time. Representative still images at the indicated time points are shown (2500 cells size). Scale bar, 100μm. Centre, schematic illustration of spheroid fusion (top), indicating the main geometrical parameters like the spheroid size R and the neck radius r. Time evolution of the normalised neck radius squared $(r/R{)}_{\infty }^{2}$ (bottom) for the samples shown in the snapshots. The dashed lines are exponential fits to the data. Right, size-dependence of the fusion rate Γ (top) and amplitude $(r/R{)}_{\infty }^{2}$ (bottom). The dashed lines represent linear fits to the data. Data are mean ± SD (n=9 SCR-500, 7 IRSp53_KD-500, 7 SCR-2500, 9 IRSp53_KD-2500, 7 SCR-5000, 8 IRSp53_KD-5000 pairs of spheroids from 2 independent experiments). **C.** Left, doxycycline-treated MCF10 DCIS.COM control (SCR) or IRSp53 silenced (IRSp53_KD) cells were cultured in ultra-low adhesion condition to form spheroids in the absence or in the presence of 0.3mM EDTA (EDTA0.3) as indicated. Pairs of spheroids were then mixed, and fusion was monitored over time. Representative still images at the indicated time points are shown. Scale bar, 100μm. Right, time evolution of the normalised neck radius $(r/R{)}_{\infty }^{2}$ (top) for the samples shown in the snapshots. Lines are exponential fits to the data as indicated. Bottom, Fusion rate Γ and amplitude $(r/R{)}_{\infty }^{2}$ for the different treatments. Data are mean ± SD (n=6 SCR, 5 IRSp53_KD, 7 SCR EDTA, 8 IRSp53_KD EDTA pairs of spheroids from 2 independent experiments). Statistical analysis for each experiment is included in the Methods section. P values are indicated in each graph.

Firstly, we measured bulk rheological properties of DCIS cells in 3D aggregates by flowing spheroids into a microfluidic device. This device is composed of a channel that presents a set of consecutive constrictions of 40μm in width alternated with wider spaces (a detailed description of the system is in B.G. and G.S., manuscript in preparation). An identical number of cells is seeded in low attachment to generate spheroids of about 100μm that are pushed to enter the constricted channels by a constant pressure and flow. The passage into the first constriction is the rate-limiting step as the spheroid must undergo a sufficient deformation to enter the channel that depend on its bulk viscoelastic properties. The deformed spheroids have insufficient time to recover their shape and rapidly pass into subsequent constricted channels. The transit time across the first constriction, which is an indirect probe of the viscous component of the spheroid rheology, was reduced by about 50% in IRSp53 KD as compared to control spheroids (Fig. 3A and Supplementary Movie S10).

This finding indicates that silencing of IRSp53 results in faster spheroid deformation, consistently with a reduction in tissue viscosity and increased fluidization even in a much higher frequency regime compared to the one probed in spreading experiments.

Secondly, we conducted spheroid-fusion experiments. In these experiments, two spheroids of the same cell type and similar radius $R_{0}$ are put in contact, and their evolution over time is monitored. In close analogy with the behavior of two viscoelastic droplets, we observed a progressive fusion of the spheroids, which we monitored by considering the growth of the angle radius r(t) of the “neck” connecting the fusing spheroids (Fig. 3B). For fluid droplets, the coalescence process is driven by the surface tension σ of the fluid, which tends to minimize the interfacial area, and it is slowed down by the viscosity η, which hinders fluid flow. The initial fusion rate for two droplets of radius $R_{0}$ is $\Gamma ≃\sigma /\eta R_{0}$^35^ (Fig. 3B). The presence of a non-negligible elastic component in the fluid rheology can prevent the complete fusion into a single spherical droplet. In general, the aspect ratio of the final aggregate is expected to be an increasing function of both the ratio between elastic modulus and surface tension and the initial radius $R_{0}$ of the coalescing droplets^36^.

For both control and IRSp53-devoid spheroids, the fusion rate Γ, obtained by fitting a simple exponential function $a\left(1-e^{-\Gamma t}\right)$ to the normalized variable $(r/R{)}^{2}$, displayed a linear scaling with the inverse of the spheroid radius, as expected based on a viscoelastic droplet model. The amplitude term $a=(r/R{)}_{\infty }^{2}$ is expected to be ≃1 for complete fusion, while for arrested coalescence a<1. A linear fitting of $\Gamma^{-1}\left(R_{0}\right)$ enabled estimating the ratio σ/η between effective surface tension and viscosity for both IRSp53 KD $\left(\sigma /\eta =(1.1\pm 0.2)\cdot 10^{-1}{\;\text{ms}}^{-1}\right)$ and control spheroids $\left(\sigma /\eta =(5.7\pm 1)\cdot 10^{-2}{\;\text{ms}}^{-1}\right)$. Notably, IRSp53-devoid spheroids fused faster (Fig. 3B), leading to significant increase (roughly by a factor of two) of the estimated ratio between surface tension and viscosity. Moreover, while small spheroids fused almost completely $\left((r/R{)}_{\infty }^{2}≃1\right)$, for the largest spheroids, we observed arrested coalescence, the deviation of the final configuration from a single sphere being stronger for control spheroids compared to IRSp53 KD spheroids of the same size (Fig. 3B).

Importantly, treating spheroids with EDTA, which sequesters ions crucial for preserving the integrity of cell-cell junctions^37^—whose disruption leads to a decrease in tissue viscosity^38,39^—resulted in a fusion rate increase of the spheroids comparable to that observed with the loss of IRSp53 (Fig. 3C). Moreover, EDTA-treated spheroids displayed complete coalescence $\left((r/R{)}_{\infty }^{2}≃1\right)$, while this is not the case for control spheroids $\left((r/R{)}_{\infty }^{2}≃0.80\pm 0.05\right)$ (Fig. 3C), suggesting that the treatment also reduces bulk elasticity. Notably, the combined loss of IRSp53 and EDTA treatment further increased the fusion rate and led to the complete fusion of the spheroids. These findings support the idea that the loss of IRSp53 changes the biophysical properties of spheroids by decreasing viscosity and promoting tissue fluidization.

### IRSp53 links individual cell mechanics to overall tissue tension to control wetting transition

In addition to decreased viscosity and increased fluidification, IRSp53 may also impact the effective surface tension $\left(\sigma \right)$ of cell aggregates. This analysis is essential for distinguishing the various factors that contribute to the observed increased fusion rate in IRSp53 knockdown spheroids.

The effective surface tension σ of a cell aggregate represents the ratio between the energetic cost of a deformation leading to a change ΔA in the surface area (cell-matrix contact area) and the area change itself. Within the framework of the differential adhesion hypothesis (DAH), σ is determined by the cell-cell adhesion energy per unit area Γ,^40^

(5) $$\sigma ≃\frac{1}{2}\Gamma .$$

Physically, this equality relays on the simplifying assumption that the cortical tension $\beta_{cc}$ in portions of the cell membrane corresponding to cell-cell contacts does not significantly differ from the cortical tension $\beta_{cm}$ associated with cell-matrix contacts^40^.

**Figure 4.**
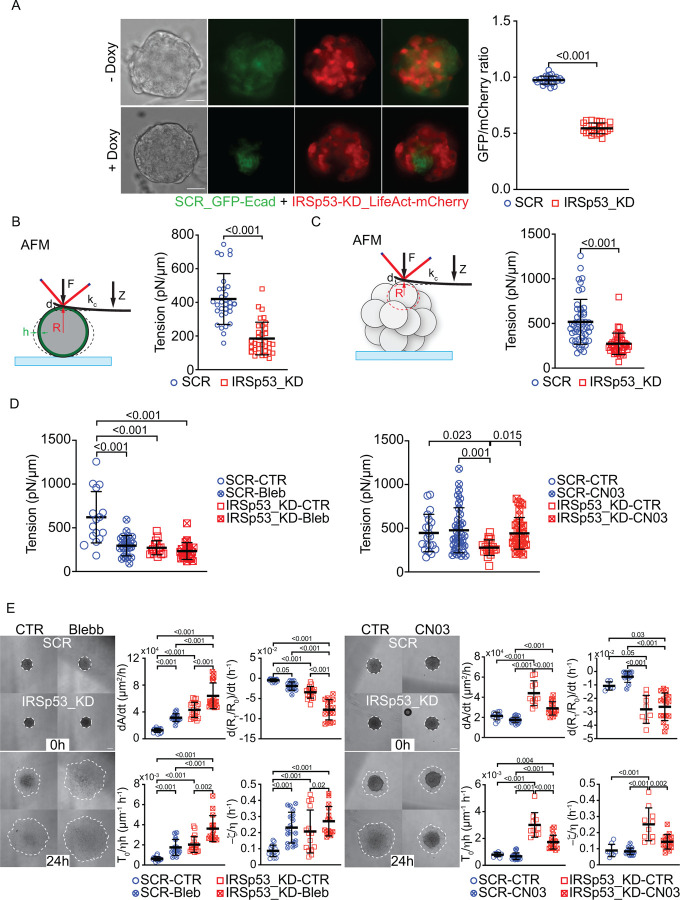
IRSp53 links individual cell mechanics to overall tissue tension to control wetting transition. **A.** Spheroids sorting. MCF10 DCIS.COM control (SCR) expressing GEP-E-Cad or IRSp53 silenced (IRSp53_KD) expressing mCherry-LifeAct not treated (−Doxy) or treated (+Doxy) cells were mixed 1:1 and cultured in ultra-low adhesion condition to form spheroids. 48h after seeding, live spheroids were visualized with EVOS cell imaging system. Left, representative bright fields and fluorescent images are shown. Scale bars, 200μm. Right graph, analysis of the GFP/mCherry signal area ratio, as a proxy of the cell sorting, was performed on mixed spheroids derived from control (SCR) expressing GFP-H2B or IRSp53 silenced (IRSp53_KD) expressing mCherry-H2B cells spheroids (not shown), by determining fluorescent area contour with ImageJ. Data are mean ± SD (n=27 −Doxy, 26 +Doxy mixed spheroids from 3 independent experiments). **B.** Mechanical probing of weakly adherent single cells by AFM. Left, schematic representation of a nonadherent cell being slightly deformed by a tipless AFM cantilever. When deforming the spherical cell, changes in laser location in the photodetector are acquired and transformed to deflection in length units. F is the applied normal force, d is the cantilever deflection, Z is the piezo movement, ${\text{k}}_{\text{c}}$ is the cantilever spring constant, R is the initial cell radius, and h is the cortical actin thickness^44^. Right, cortical actomyosin tension of doxycycline-treated MCF10 DCIS.COM control (SCR) or IRSp53 silenced (IRSp53_KD) cells extracted at a Z-piezo distance no greater than 1000nm. Data are mean ±SD (n=36 cells from 3 independent experiments). **C.** Left, schematic representation of a weakly adherent spheroids being slightly deformed by a tipless AFM cantilever. When deforming the apical cell, changes in laser location in the photodetector are acquired and transformed to deflection in length units. F is the applied normal force, d is the cantilever deflection, Z is the piezo movement, ${\text{k}}_{\text{c}}$ is the cantilever spring constant and R is the initial cell radius^44^. Right, cortical actomyosin tension of doxycycline-treated MCF10 DCIS.COM control (SCR) or IRSp53 silenced (IRSp53_KD) cells in the context of 500 cells spheroids extracted at a Z-piezo distance no greater than 1000nm. Data are mean ±SD (n=48 SCR, n=42 IRSp53_KD cells from 3 independent experiments). **D.** Left, cortical actomyosin tension of doxycycline-treated MCF10 DCIS.COM control (SCR) or IRSp53 silenced (IRSp53_KD) cells in the context of 500 cells spheroids, treated with DMSO or 5μm Blebbistatin, extracted at a Z-piezo distance no greater than 1000nm. Data are mean ±SD (n=16 SCR, n=24 SCR+Blebb, n=16 IRSp53_KD, n=24 IRSp53_KD+Blebb cells from 3 independent experiments). Right, cortical actomyosin tension of doxycycline-treated MCF10 DCIS.COM control (SCR) or IRSp53 silenced (IRSp53_KD) cells in the context of 500 cells spheroids, treated with vehicle or 10μg/ml CN03, extracted at a Z-piezo distance no greater than 1000nm. Data are mean ±SD (n=20 SCR, n=35 SCR+CN03, n=20 IRSp53_KD, n=35 IRSp53_KD+ CN03 cells from 3 independent experiments). **E.** Left, Doxycycline-treated MCF10 DCIS.COM control (SCR) or IRSp53 silenced (IRSp53_KD) cells were cultured in ultra-low adhesion condition to form spheroids. 6h before seeding for spreading, spheroids were added with DMSO (CTR) or 5μm Blebbistatin. Spheroids were then seeded on fibronectin coated 6 well plates and spreading monitored by time-lapse microscopy (Supplementary Movie S11). Left, Representative still images at the indicated time points are shown on the left. Scale bar, 200μm. Right, spreading parameters obtained from image segmentation and PIV analysis: rate of spreading dA/dt, normalised rate of core melting d(R1/R0)/dt, ratio between traction and viscosity, A and B. Data are mean ± SD from 3 independent experiments (dA/dt: n=15 SCR, 15 SCR-Bleb, 21 IRSp53_KD, 18 IRSp53_KD-Bleb spheroids; d(R1/R0)/dt, A and B: n=11 SCR, 13 SCR-Bleb, 20 IRSp53_KD, 18 IRSp53_KD-Bleb spheroids. **F.** Left, doxycycline-treated MCF10 DCIS.COM control (SCR) or IRSp53 silenced (IRSp53_KD) cells were cultured in ultra-low adhesion condition to form spheroids. 6h before seeding for spreading, spheroids were added with vehicle (CTR) or 10μg/ml CN03. Spheroids were then seeded on fibronectin coated 6 well plates and spreading monitored by time-lapse microscopy (Supplementary movie S12). Representative still images at the indicated time points are shown. Scale bar, 200μm. Right, spreading parameters obtained from image segmentation and PIV analysis. From top to bottom: rate of spreading dA/dt, normalised rate of core melting d(R1/R0)/dt, ratio between traction and viscosity, ratio between contractility and traction. Data are mean ± SD from 3 independent experiments (dA/dt: n=11 SCR, 19 SCR-CN03, 10 IRSp53_KD, 16 IRSp53_KD-CN03 spheroids; d(R1/R0)/dt: n=6 SCR, 11 SCR-CN03, 8 IRSp53_KD, 16 IRSp53_KD-CN03 spheroids; A and B: n=8 SCR, 16 SCR-CN03, 10 IRSp53_KD, 16 IRSp53_KD-CN03 spheroids. Statistical analysis for each experiment is included in the Methods section. P values are indicated in each graph.

We obtained direct evidence that IRSp53 significantly impacts the surface tension of cell aggregates employing demixing experiments of cells in 3D spheroids composed of genetically different cells. When SCR control and IRSp53-deficient MCF10.DCIS.com cells were mixed in a 1:1 ratio and allowed to form spheroids, IRSp53-deficient cells preferentially migrated to the outer layer, while SCR cells remained in the core (Fig. 4A). According to the DAH, cells forming aggregates with lower surface tension—due to weaker intercellular adhesion—tend to localize at the periphery of a tissue, whereas cells with stronger adhesion remain in the core^41,42^. The observed sorting pattern clearly indicates that IRSp53-deficient cells differ in their cell adhesion.

Tissue mechanical properties are intrinsically linked to the mechanical behavior and state of individual cells^2,43^. Therefore, the global changes observed upon IRSp53 loss likely reflect its role in modulating individual cell mechanics^14–16,31^. Consequently, we investigated the impact of IRSp53 on cortical tension in both isolated cells and within large 3D aggregates using various approaches.

One approach involved atomic force microscopy (AFM) using a tipless cantilever^44^ to measure cortical tension in poorly adherent MCF10 DCIS.com single cells. The tipless cantilever allows for precise measurement of cell mechanical properties without piercing the cell membrane, focusing on the cortical tension $\beta_{cm}$ as a key indicator of mechanical changes^44^. We found that IRSp53 loss reduced cell cortical tension by 50% (Fig. 4B). Similar results were also obtained by probing with the tipless cantilever individual cells in the context of 3D-spheroids (Fig. 4C). To confirm that the loss of IRSp53 affects cortical tension via actomyosin contractility, we tested cells under conditions where contractility was pharmacologically perturbed. Inhibition of actomyosin contractility with Blebbistatin resulted in a similar reduction in cortical tension as observed with IRSp53 knockdown, suggesting that both conditions disrupt tension in comparable ways (Fig. 4D). Conversely, promoting actomyosin contractility through RhoA activation with CN03 rescued the cortical tension defects in IRSp53-deficient cells, restoring their mechanical properties (Fig. 4D).

These findings demonstrate that IRSp53 modulates actomyosin-mediated cell tension, a critical factor in wetting transition dynamics alongside tissue viscosity, as predicted by our biophysical model. Actomyosin contractility is essential not only for regulating cell tension but also for maintaining global cell cohesion during wetting in IRSp53-depleted cells. This is evidenced by spheroid spreading rates, where Blebbistatin accelerated spreading and CN03 reduced it (Fig. 4E–F, Supplementary Fig. S2C–F and Supplementary Movies S11–12).

**Figure 5.**
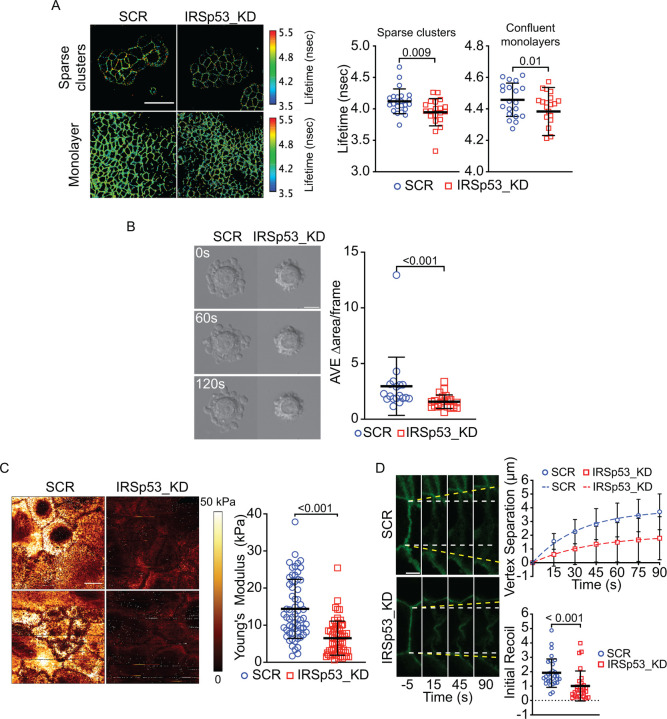
**A.** Live confocal FLIM analysis of doxycycline-treated SCR and IRSp53_KD MCF10 DCIS.COM cells seeded to obtain sparse cell clusters or at jamming density. 72h after seeding cells were added with Flipper-TR fluorescent membrane tension probe and analysed by confocal microscopy. Left, representative FLIM images with the corresponding calibration bars representing LUT of average Flipper-TR fluorescence lifetime expressed in nanoseconds (nsec). Scale bar, 10μm. Right graphs, quantification of in vivo changes in membrane tension of cells clusters or confluent monolayers respectively. Data are mean ± SD (n=23 SCR, 20 IRSp53_KD cells in sparse clusters and n=20 SCR, 19 IRSp53_KD cells in confluent monolayers from 2 independent experiments). **B.** IRSp53 removal reduces cell blebbing after detachment. Time-lapse microscopy (5 min, time frame 10 sec) of doxycycline-treated SCR and IRSp53_KD MCF10 DCIS.COM detached cells 5 min after treatment with trypsin and gentle pipetting (Supplementary Movie S13). Left, representative still images at the indicated time points are shown. Scale bars, 10μm. Right, blebbing activity was analysed as cell area variation over time (Δarea/frame) by XXX software. Data are mean ± SD (n= 18SCR, 24 IRSp53_KD cells from 2 independent experiments). **C.** Atomic force microscopy analysis of doxycycline-treated SCR and IRSp53_KD MCF10 DCIS.COM cells seeded and grown to full confluency. Left, representative maps of Quantitative Imaging to collect Young’s modulus maps of regions of cells 50×50 square microns in area. Scale bar, 10μm. Right, Young’s modulus quantification. Data are mean ± SD (n=62 fields from 3 independent experiments). **D.** Left, image sequence of GFP-CaaX-positive junctional vertices recoiling after nano-scissor laser ablation at t=0 in SCR and IRSp53_KD cells (Supplementary movie S14). Recoiling vertices were measured as a proxy of junctional tension. White dashed lines indicate starting positions of vertices; yellow dotted lines indicate expansion of vertices after laser ablation. Scale bar, 5μm. Top right graph, initial recoil was measured by the instantaneous rate of vertex separation at t=0 and computed using best fit single exponential curves. Bottom right plot, initial recoil rate. Data are the means ± SD, normalized to control. Data are the means ± SD (n=24 SCR, 30 IRSp53_KD from 2 independent experiments). Statistical analysis for each experiment is included in the Methods section. P values are indicated in each graph.

The cortical actin network is tightly coupled to the plasma membrane and plays a crucial role in maintaining membrane tension^45,46^, such that changes in cortical tension are often reflected at the membrane level. Thus, we next measured membrane tension to explore how cortical tension reduction might extend to the plasma membrane. We use FLIM microscopy with the Flipper-TR reporter^47^ that allows to visualize and measure changes in plasma membrane tension in real time^48^. We observed that IRSp53 ablation led to a significant reduction in membrane tension, both in sparsely distributed cell clusters and in confluent monolayers (Fig. 5A), further supporting its role in regulating both cortical and membrane mechanics.

Cortical and membrane tension are critical parameters that govern the ability of isolated cells to form blebbing like protrusions either under confinement or when detached and left in suspension. We utilized this latter approach to monitor the blebbing capacity of detached, isolated cells^49^. Under such conditions, IRSp53 downregulation robustly reduced blebbing activity of single cells (Fig. 5B and Supplementary Movie S13), strengthening the notion that this protein is essential in regulating single-cell mechanical properties.

We also performed high resolution Atomic Force Microscopy nano-mechanical mapping. We observed that the loss of IRSp53 significantly reduces global apical and junctional stiffness, as measured by a drop in Young’s modulus in confluent monolayers (Fig. 5C).

The reduced intercellular and apical stiffness suggests that also junctional tension may be reduced following the loss of IRSp53. We tested this directly by measuring the rate of vertex recoiling after two-photon laser mediated junctional nano-scission. The instantaneous recoil from the cut site indicated that IRSp53 ablation decreased local junctional tension compared to control cells (Fig. 5D and Supplementary Movie S14).

Altogether, these findings demonstrate that IRSp53 coordinates global tissue tension by regulating the mechanical properties of individual cells, thereby controlling the wetting transition in multicellular spheroids.

### IRSp53 regulates viscosity and surface tension by controlling cell junction architecture and interfacial junctional tension

How does IRSp53 control spheroid viscosity and tension? The viscosity of a cell aggregate depends on both intracellular interactions and the mechanical properties of individual cells. Intercellular tension arises from the balance of forces generated by cellular adhesion, the cytoskeleton, and intracellular contractility. It is further influenced by the structural architectural organization of cell-cell junction that directly impinges on the ability to transmit long-range forces and generate interfacial cell tension. We tested how IRSp53 impacts these properties using several orthogonal approaches.

**Figure 6.**
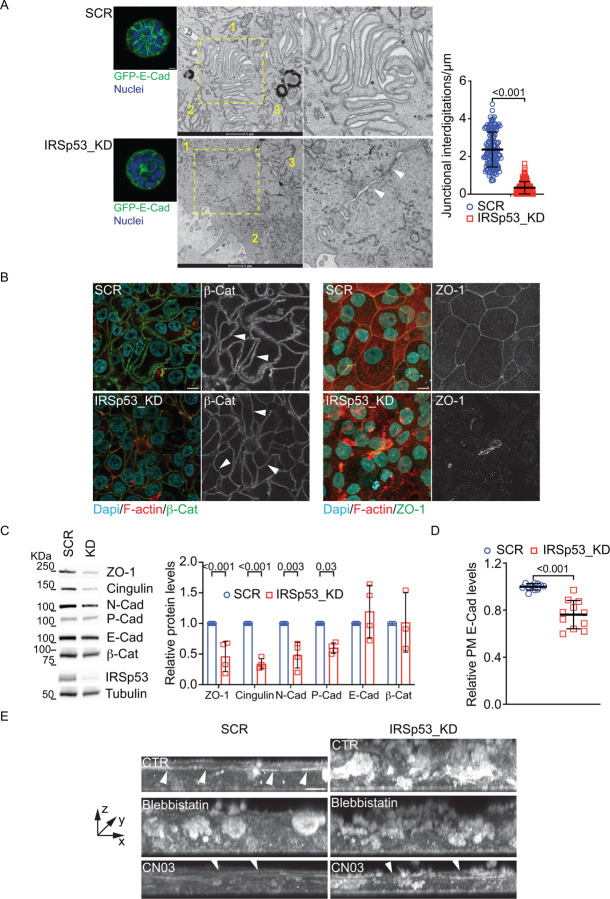
IRSp53 regulates viscosity and surface tension by controlling cell junction architecture. **A.** Left, doxycycline-treated MCF10 DCIS.COM control (SCR) or IRSp53 silenced (IRSp53_KD) cells expressing GFP-E-Cad were seeded as single cells on Matrigel-coated gridded coverslips and cultured in medium containing 2% Matrigel. The deriving spheroids were stained with NucBlue^™^ Live ReadyProbes ^™^ (nuclei, blue) and identified on grids by confocal microscopy. Scale bar, 10μm. Samples were then processed for electron microscopy and images of the corresponding spheroids are shown. Numbers indicate singles cells within the spheroids. Right panels represent 2x magnification of the area depicted by the white dashed squares. Arrowheads indicate the loss of plasma membrane interdigitations at cell-cell junction in IRSp53_KD samples. Right, quantification of junctional interdigitation/μm. Data are mean ± SD (n=152 fields/4 spheroids CTR, 204 fields/4 spheroids IRSp53_KD from 3 independent experiments. Samples were counted from serial sections along the Z-axis. FIB-SEM tomography was performed on the same spheroids (Supplementary Movies S15–S16). **B.** Left, confocal microscopy image sections (5μm from the bottom adherent surface) of doxycycline-treated SCR and IRSp53_KD MCF10 DCIS.COM cells seeded at jamming density and fixed 96 hours after seeding. Cells were stained with anti-β-catenin antibody (green), TRITC-phalloidin to detect F-actin (red), and DAPI (blue). Right, arrowheads indicate the different architecture and thinning of cell-cell junctions highlighted by β-catenin staining. Scale bar, 10μm. Right, confocal microscopy analysis of doxycycline-treated SCR and IRSp53_KD MCF10 DCIS.COM cells seeded at jamming density and fixed 96 hours after seeding. Cells were stained with anti-ZO-1 antibody (green), TRITC-phalloidin to detect F-actin (red), and DAPI (blue). Confocal section on z axis were acquired with 0.5μm step-size. Representative maximum projection images are shown. Right, ZO-1 staining is shown. Scale bar, 10μm. **C.** IRSp53 removal perturbs junctional protein levels. Doxycycline-treated SCR or IRSp53_KD MCF10 DCIS.COM cells were seeded at jamming density and processed for WB analysis 4 days after seeding. Left, WB analysis, with the indicated antibodies, to detect junctional proteins and IRSp53 levels. Right, relative protein levels analysis (normalized on Tubulin levels) was determined with ImageJ. Data are mean ± SD (n=4 independent experiments ZO-1, Cingulin, E-Cad, P-Cad, N-Cad, 3 independent experiments β-Cat). **D.** IRSp53 loss alters E-Cad plasma membrane levels. FACS analysis of cell surface E-cadherin (E-Cad) in control (SCR) and IRSp53-silenced (IRSp53_KD) MCF10 DCIS.COM cells. Data are represented as mean fluorescence intensity, fraction of SCR ± SD (n=11 technical replicates from n=5 independent experiments). **E.** Confocal reconstruction of doxycycline-treated SCR and IRSp53_KD MCF10 DCIS.COM cells seeded at jamming density and fixed 96 hours after seeding. 16h before fixation, cells were treated with DMSO (CTR), 5μm Blebbistatin or 10μg/ml CN03. After fixation, cells were stained with anti-β-Catenin (not shown) or anti-ZO1 (not shown) antibodies, TRITC-phalloidin to detect F-actin, and DAPI (not shown). Confocal section on z axis were acquired with 0.5μm step-size and resliced on y axis using ImageJ. Maximum projections of the F-actin channels are shown. Arrowheads (SCR CTR, SCR CN03 and IRSp53_KD CN03) indicates the supracellular actin cable. Scale bar, 10μm. Statistical analysis for each experiment is included in the Methods section. P values are indicated in each graph.

Firstly, we perform Correlative Light and Electron Microscopy (CLEM) analysis of cell aggregates stably expressing GFP-E-Cadherin to visualize cell junctions. This analysis revealed that loss of IRSp53 resulted in the formation of architecturally aberrant cell-cell junctions (Fig. 6A). Two recent studies showed that epithelial adherens junctions in monolayers are not entirely flat but appear to contain interdigitated actin-rich microspikes that contribute to cell–cell adhesion^19,20^. Similarly, we detected extended interdigitations in control MFC10 DCIS.com spheroids, which instead were nearly absent in 3D cell aggregates devoid of IRSp53 (Fig. 6A). FIB-SEM tomographic reconstruction further confirmed the altered interdigitated structures at cell junction of IRSp53 KD spheroids (Supplementary Movies S15–16).

The alterations of junctional architecture were also highlighted by immunofluorescence confocal microscopy of adherens junctions, using β-catenin, and tight junctions, marked by ZO-1 (Fig. 6B) staining, in MCF10 DCIS.com monolayers. Indeed, the adherens junctions were thinner in IRSp53 KD-epithelial layers, while a global reduction in ZO-1 and its drastic reduction along cell junctions indicated the absence of an organized network of tight junctions.

The altered structural organization with the loss of interdigitating intercellular contacts suggests that the strength of cell-cell adhesion might be weakened following the silencing of IRSp53. In keeping with this possibility, IRSp53 KD MCF10.DCIS.com monolayers display frequently extended and aberrantly enlarged intercellular spaces (Supplementary Fig. S3A and Supplementary Movies S17–18). Notably, the aberrant architectural organization of junctions in IRSp53 devoid cells was also accompanied by a general reduction in the overall expression of several junctional markers, including ZO-1, Cingulin, N-Cadherin and P-Cadherin (Fig. 6C) and a slight but significant reduction of cell surface E-Cadherin (Fig. 6D).

Epithelial cells after prolonged culture as 2D monolayers have been shown to spontaneously self-organized to generate the formation of dynamics dome-like structures on solid growth supports. Dome formation requires active polarized fluid transport and the establishment of tight supracellular barrier and tensile junctional structures^50^, which were compromised in IRSp53 silenced monolayers. Consistently, control monolayers formed dynamically expanding and contracting domes, which were instead completely absent after silencing of IRSp53 (Supplementary Fig S3B–C and Supplementary Movie S19–20).

The altered junctional architecture may also compromise the junctional tensile state and prevent the formation of supracellular tensile cytoskeletal structures. In line with this, confocal analysis of MCF10 DCIS.com monolayers revealed that IRSp53 removal impeded the formation of force transmitting actin cables that in control cells extend through cell adhesive junction across multiple cells (Fig. 6E). Notably, the formation of supracellular actin cable in IRSp53 silenced cells could be nearly completely rescued by increasing actomyosin contractility by treatment of monolayers with the RhoA-activator CN03 (Fig. 6E). Conversely, the inhibition of actomyosin contractility by addition of Blebbistatin to control monolayers disrupted the formation of a supracellular actin cable phenocopying the loss of IRSp53 (Fig. 6E).

Thus, IRSp53 controls tissue viscosity and surface tension by regulating cell junction structure and interfacial tension, reinforcing the notion that this protein is crucial in linking global tissue mechanics to the mechanical properties of individual cells.

### IRSp53 interact with Afadin to control viscoelasticity.

**Figure 7.**
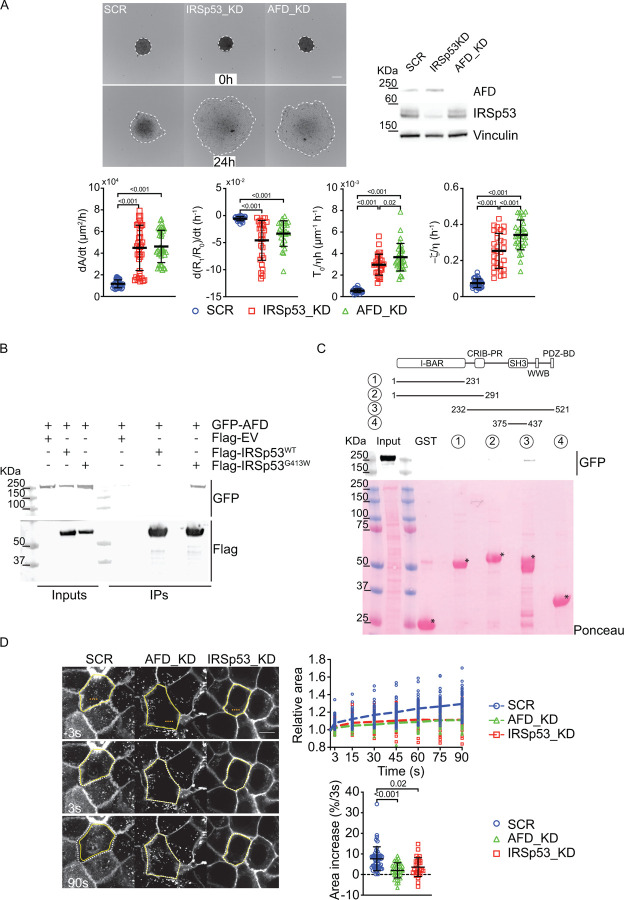
IRSp53 interacts with Afadin to control viscoelasticity. **A.** Doxycycline-treated MCF10 DCIS.COM control (SCR), IRSp53 silenced (IRSp53_KD) or Afadin silenced (AFD_KD) cells were cultured in ultra-low adhesion condition to form spheroids. Spheroids were then seeded on fibronectin coated 6 well plates and spreading monitored by time-lapse microscopy (Supplementary Movie S21). Upper left, representative still images at the indicated time points are shown. Scale bar, 200μm. Upper right, WB analysis, with the indicated antibodies, was performed to detect IRSp53 and Afadin levels. Lower graphs, spreading parameters obtained from image segmentation and PIV analysis. From left to right: rate of spreading dA/dt, rate of core melting d(R1/R0)/dt, A and B. Data are means ± SD from 3 independent experiments (dA/dt: n=34 SCR, 37 IRSp53_KD, 36 AFD-KD spheroids; d(R1/R0)/dt: n=26 SCR, 29 IRSp53_KD, 26 AFD-KD spheroids; A and B: n=34 SCR, 31 IRSp53_KD, 33 AFD-KD spheroids). **B.** IRSp53 interaction with Afadin is enhanced by loss of function of the SH3 domain. Co-immunoprecipitation: lysates (4 mg) of 293T cells, transfected with the indicated constructs, were immunoprecipitated with an anti-Flag antibody. Input lysates (20μg) and immunoprecipitates (IPs) were immunoblotted with the indicated antibodies. **C.** Analysis of IRSp53-Afadin surface interaction. Lysates (2 mg) of 293T cells transfected with GFP-Afadin were incubated with 0.5μM of immobilized GST–IRSp53 fragment as indicated or GST. Lysates (20μg) and bound proteins were immunoblotted with anti-GFP antibody. Ponceau staining was employed to detect GST recombinant proteins. **D.** IRSp53 loss decrease cell cortical tension. Doxycycline-treated MCF10 DCIS.COM control (SCR), Afadin silenced (AFD_KD) or IRSp53 silenced (IRSp53_KD) cells were cultured as monolayers. Left, examples of terminal web laser ablation in SCR, AFD_KD and IRSp53_KD cells expressing, mCherry-CaaX (see also Supplementary Movie S22). Location of ablation is depicted by dotted orange line. The cell contour at t = 0 is represented by a full yellow line (kept on all images), and the one at later time points is represented by a dashed with line. Scale bar, 10μm. Upper right graph, quantification of the evolution of the normalized apical surface area over time after ablation in SCR, AFD_KD and IRSp53_KD cells. Geometric shapes, individual cells, dashed lines averaged over one cell type. For normalization, all cell areas are scaled to 1 at t = 0. Bottom right graph, relative value of initial area spreading measured as the relative area difference over time between t = 0 and t = 3s^29^. Data are mean ± SD (n=50 SCR, 36 AFD_KD, 29 IRSp53_KD cells from 2 independent experiments). Statistical analysis for each experiment is included in the Methods section. P values are indicated in each graph.

IRSp53 is a multidomain protein that comprises: a membrane-binding BAR domain followed by an unconventional CRIB motif, which overlaps with a proline-rich region (CRIB–PR); an SH3 domain that recruits actin cytoskeleton effectors; a PDZ binding motif at the very C-Term^14,15,51^. We investigated the IRSp53 proximity interactome, previously generated using both canonical and quantitative SILAC proteomics combined with BirA-based proximity labeling^21^. This approach allowed us to identify proteins in close proximity or binding to IRSp53, providing insights into its interaction network. Many of the newly identified interactors were cell junction proteins. Through RNAi screening in a 3D spheroid spreading assay, we tested multiple candidate genes to determine which interactors of IRSp53 could mimic its loss-of-function phenotypes. Afadin (AFD) emerged as the only effective candidate. Notably, Afadin is a junctional protein essential during development in mice, *Drosophila* and *Caenorhabditis elegans*^26–28^; it has been shown to specifically influence the epithelial cortical tension^29^ and the epithelial polarity program as IRSp53^30^. This suggests that Afadin may function in a similar pathway or as a key partner in regulating cell junctions and collective cell movement, making it a critical player in maintaining tissue integrity alongside IRSp53. Consistently, Afadin ablation phenocopied IRSp53 loss during 3D-spheroids wetting (Fig. 7A, Supplementary Fig. S3D–E and Supplementary Movie S21). To confirm the IRSp53-Afadin interaction, we performed co-immunoprecipitation experiments and found that ectopically expressed Afadin binds to the IRSp53-SH3 mutant (W413G)^16^ but not the wild-type form (Fig. 7B). This suggests that the SH3 domain normally inhibits Afadin binding in the wild-type protein. IRSp53 is known to fold into a closed, inactive conformation, with the CRIB-PR region binding the SH3 domain. Activation requires cooperative interactions: Cdc42 binding to its CRIB-PR domain and effector proteins, such as Eps8, binding to the SH3 domain, open the protein and expose interaction sites^51^. Hence, Afadin likely interacts with the open conformation, independent of the SH3 domain. *In vitro* pull-down assays with purified IRSp53 fragments confirmed that Afadin binding requires the C-terminal region of IRSp53, not the SH3 domain (Fig. 7C). This reinforces that the SH3 domain regulates IRSp53’s conformation and interactions^51^, while Afadin associates specifically with the C-terminal region when IRSp53 is in its active, open form.

Afadin (AFD) has been identified as critical for maintaining the epithelial cell cortical tension by regulating the apical contraction apparatus^29^. To investigate whether IRSp53 influences cortical tension in a similar manner, we conducted apical surface laser ablation experiments following IRSp53 depletion. The results showed that the loss of IRSp53 led to a reduction in apical tension, mimicking the effects seen with Afadin depletion (Fig. 7D, Supplementary Movie 22). This confirms that both IRSp53 and Afadin collaborate to regulate epithelial mechanics and maintain tissue integrity by controlling cortical tension. Their functional interaction at cell junctions highlights their role in shaping tissue architecture, especially in the dynamic regulation of mechanical forces across epithelial layers.

## Conclusion and perspective

This study establishes IRSp53 and its functional partner Afadin as central regulators of the viscoelastic properties of epithelial tissues, providing a molecular framework for understanding the solid-to-fluid transitions essential to tumor progression. We demonstrate that IRSp53 exerts its effects by modulating junctional architecture, tensile state, and intercellular cohesion, processes that are directly linked to tissue viscosity and fluidification. The depletion of IRSp53 reduces supracellular tension and disrupts the structural organization of adherens and tight junctions, leading to decreased intercellular friction and enhanced local cell rearrangements. This drives a shift from a solid-like to a fluid-like state, evidenced by increased tissue spreading rates and spheroid fluidization in both 2D monolayers and 3D spheroids.

At the molecular level, IRSp53’s interaction with Afadin emerges as a critical mechanism underlying these transitions. Afadin, known to regulate cortical tension and epithelial polarity, collaborates with IRSp53 to maintain the integrity of cell-cell junctions and supracellular tensile structures. Together, they bridge cellular mechanics with tissue-scale dynamics, enabling the regulation of active wetting and phase transitions that underpin epithelial tissue remodeling during cancer progression. This interplay highlights how cellular-scale processes, such as junctional tension and actin cytoskeleton organization, drive emergent tissue behaviors, including viscosity modulation and collective cell motility.

The findings of this study contribute to a growing understanding of the molecular determinants of tissue mechanics in cancer biology. By revealing the critical roles of IRSp53 and Afadin in regulating tissue fluidification, this work paves the way for potential novel therapeutic strategies targeting solid-to-fluid transitions in cancer. Future studies however are necessary to explore how these molecular pathways integrate with other biophysical processes, such as extracellular matrix remodeling and immune cell infiltration, to drive tumor progression and metastasis. Furthermore, expanding the analysis of IRSp53 and Afadin functions to other tissue types and disease models could uncover broader implications for their roles in development, regeneration, and disease.

## Methods

### Antibodies and DNA constructs

The complete list of used antibodies and DNA constructs is reported in the “Reagents List” table.

### Cell culture

MCF10 DCIS.com cell line was cultured at 37°C in humidified atmosphere with 5% CO2 in Dulbecco’s Modified Eagle Medium (DMEM):Nutrient Mixture F-12 (DMEM/F12) GlutaMAX medium (Life Technologies) supplemented with 5% horse serum, 0.5mg/mL hydrocortisone, 10mg/mL insulin, and 20ng/mL EGF. HaCat cell line was cultured at 37°C in humidified atmosphere with 5% ${\text{CO}}_{2}$ cultured in DMEM High Glucose medium (Euroclone) supplemented with 10% Fetal Bovine Serum and 4mM L-Glutamine.

To treat monolayer and wetting spheroids, blebbistatin (B0560 - Sigma-Aldrich) and RhoA Activator II (CN03A – Cytoskeleton) were added ~6 hours before imaging and respectively used at the final concentration of 5mM and 10μg/ml.

### IRSp53 knock-down & over-expression

To perform IRSp53 ablation in MCF10 DCIS.com and HaCat cells, we cloned a specific shRNA against human IRSp53 into Tet-pLKO-puro plasmid (Addgene Plasmid #21915). The target sequence was identified with the online tool from Broad RNAi Consortium (https://portals.broadinstitute.org/gpp/public/seq/search) and is the following: CAGCAAGAATCCTCAGAAGTA. As negative control, we used a Scrambled (shSCR) sequence (GTGGACTCTTGAAAGTACTAT)^52^. SCR and IRSp53_KD bulk populations were obtained by lentiviral transduction and selected by puromycin (final concentration: 2μg/ml). To get IRSp53 depletion, we added 1μg/ml doxycycline to the medium 48–72 hours before starting each experiment, to allow degradation of endogenous protein. Efficiency of inducible knock-down was evaluated by Western blot. To rescue IRSp53 expression, we cloned murine IRSp53 into pRRL-SIN vector and obtained stably-expressing cells by lentiviral transduction.

In the RAB5A-dependent collective motility experiments, IRSp53 down-modulation was achieved by a specific siRNA (SASI_HS01_00210743 from Sigma-Aldrich, target sequence: GGAAGAAATGCTGAAGTCT), while a Luciferase siRNA (Duplex from Dharmacon, sequence: CATTCTATCCTCTAGAGGATG) was used as negative control. siRNAs were transfected in 2 successive cycles (reverse and forward) at the final concentration of 25nM with RNAiMAX (13778150 – Thermofisher), according to manifacturer’s instructions. Efficiency of Rab5A over-expression and IRSp53 knock-down were evaluated by qPCR with the following Taqman assays: RAB5A:hs00702360_s1 and BAIAP2:Hs00170734_m1.

To perform afadin ablation in MCF10 DCIS.com, pGFP-shLenti-AFD shRNA2 construct was stably expressed by lentiviral transduction (a kind gift from Marina Mapelli Lab^30^).

### Collective motility (wound-healing)

SCR and IRSp53_KD cells were seeded in 6-well plate (1.5*10^6^ cells/well) in complete medium with doxycycline 1μg/ml. The day after, monolayer was scratched with a pipette tip and carefully washed with 3X PBS to remove cells and create a cell-free wound area. The closure of the wound was monitored by time-lapse with Leica TIRF inverted microscope (widefield acquisition only) with 10X objective every 10 minutes for 24 hours, at 37°C in humidified atmosphere with 5% ${\text{CO}}_{2}$.

The percentage of area covered by cells over time was calculated using a custom Fiji and MATLAB code. The area covered over time was fitted with two straight lines (https://github.com/aganse/MultiRegressLines.matlab/blob/master/regress2lines.m) and the slope of the second line was used to estimate the maximal rate of coverage, after a lag phase. On the cell layers identified, velocity fields were extracted by Particle Image Velocimetry (PIV) using the Matlab mpiv toolbox (https://www.oceanwave.jp/softwares/mpiv/index.php), with an interrogation window of 60×60 um and a 25 % of overlap and a time interval of 10 minutes between two consecutives frames. The correlation length (*Lcorr*) was estimated fitting the normalized velocity spatial correlation function, along the radius r, with an exponential function $e^{-r/\text{Lcorr}}$. Directionality index was obtained by manually tracking leader cells at the migrating front with Manual Tracking plugin from Fiji and analyzing obtained trajectories with Chemotaxis Tool plugin.

### Unjamming-to-Jamming transition

SCR (H2B-GFP labelled) and IRSp53_KD (H2B-mCherry labelled) cells were seeded in confluent conditions (near jamming density) in 12-well plate (6*10^5^ cells/well) in complete medium with doxycycline 1μg/ml. The day after, transition from unjamming (fluid-like) to complete jamming (solid-like) state was monitored by time-lapse with Olympus ScanR inverted microscope with 10X objective every 10 minutes for 72 hours, at 37°C in humidified atmosphere with 5% ${\text{CO}}_{2}$.

Maps of the instantaneous cellular velocities were obtained by analyzing time-lapse phase-contrast movies of cell monolayers with Particle Image Velocimetry (PIV) using a custom MATLAB script. The interrogation windows were typically 64 or 128 pixels (44.9 or 52.3μm) on a side, with a 50% overlap between adjacent windows. For a given monolayer, time-lapse images from 8 different fields of view were simultaneously collected.

PIV analysis quantifies speed and direction of cell flow within each interrogation window. From the velocity map, we computed the instantaneous velocity of the center of mass as the average velocity $v_{CM}(t)={\left\langle v_{i}(t)\right\rangle }_{i}$, where $v_{i}(t)$ is the instantaneous velocity at time t in the i-th interrogation window and $\langle \cdot \rangle_{i}$ denotes the average over all the interrogation windows. The root mean square velocity was then quantified as $v_{RMS}(t)=\sqrt{{\left.\langle |v_{i}(t)-{\left.v_{CM}(t)\right|}^{2}\right\rangle }_{i}}$. The time evolution of $v_{RMS}$ was calculated for each single field of view. Results were then averaged, at each time point, over all fields of view of the same cell type.

### RAB5A-dependent collective motility

MCF10 DCIS.com H2B-labelled cells (pSLIK-EV & pSLIK-RAB5A)^12,53^ were subjected to 2 successive cycles of RNA interference (reverse and forward) with a specific siRNA against human IRSp53 (Luciferase siRNA was used as negative control) at the final concentration of 25nM, according to manifacturer’s instructions (RNAiMax, 13778150 – Thermofisher). Cells were seeded in 6-well plate (1.5*10^6^ cells/well) in complete medium. After 48 hours from first RNA interference cycle (reverse), 2.5μg/ml doxycycline was added to induce Rab5A expression and after additional 6 hours time-lapse was started. Collective motility was monitored by time-lapse with Olympus ScanR inverted microscope with 10X objective every 5 minutes for 24 hours, at 37°C in humidified atmosphere with 5% ${\text{CO}}_{2}$. After the end of time-lapse, cells were collected for RNA extraction and analysis of RAB5A/IRSp53 expression by qPCR.

Analysis of local and collective cell motility was performed via PIV analysis of the time-lapse phase-contrast movies. The interrogation windows were 32 pixels (41.6μm) on a side, with a 50% overlap between adjacent windows.

The instantaneous velocity of the center of mass was computed as the average velocity $v_{CM}(t)={\left\langle v_{i}(t)\right\rangle }_{i}$, where $v_{i}(t)$ is the instantaneous velocity at time t in the i-th interrogation window and $\langle \cdot \rangle_{i}$ denotes the average over all the interrogation windows. The alignment between the local velocity $v_{i}(t)$ and the collective velocity $v_{CM}(t)$ was quantified within each grid point via the parameter $a_{i}=\frac{v_{i}\cdot v_{CM}}{\left\|v_{i}\right\|\left\|v_{CM}\right\|}$, where a value $a_{i}=1\left(a_{i}=-1\right)$ indicates that the local velocity is parallel (antiparallel) to the mean direction of migration.

### 1D motility

Micro-patterns of fibronectin-coated lines (10-μm wide) were fabricated using photo-lithography^54^. The glass surface of the round coverslip (25 mm diameter) was activated with plasma cleaner (Harrick Plasma) and then coated with cell repellent PLL-g-PEG (Surface Solutions GmbH, 0.5mg/mL in 10 mM HEPES). After washing with PBS 1x and deionized water, the surface was illuminated with deep UV light (UVO Cleaner, Jelight) through a chromium photomask (JD-Photodata). Then, coverslips were coated with 10ug/ml fibronectin (1056 – Sigma Aldrich).

SCR and IRSp53_KD cells were seeded onto patterned coverslips 16 hours before starting the time-lapse (1*10^4^ cells in 2 ml of complete medium with doxycycline 1μg/ml). Linear motility along patterns was monitored with Olympus ScanR inverted microscope with 10X objective every 10 minutes for 24 hours, at 37°C in humidified atmosphere with 5% ${\text{CO}}_{2}$. To perform cell segmentation and tracking, a specific software in C++ with the OpenCV [http://opencv.willowgarage.com/wiki/] and the GSL [http://www.gnu.org/software/gsl/] libraries was developed. The analysis of cell migration was performed by the C++ software coupled with R [www.R-project.org]^55^.

### Spheroid formation & wetting

In the majority of wetting experiments, SCR and IRSp53_KD spheroids (from MCF10 DCIS.com and HaCat cells) were obtained by seeding 5*10^3^ cells in 200 ul of complete medium with doxycycline 1μg/ml in 96-well Clear Round Bottom Ultra-Low Attachment Microplate (7007 – Corning) and centrifuging them for 5 minutes at 1200 rpm, to allow the gathering of cells at central area of each well. After 16 hours, compact roundish spheroids are formed (one for each well). In specific experiments, spheroids of different sizes were obtained by seeding 2.5*10^2^, 1*10^3^ or 5*10^3^ cells according to the protocol described above.

For wetting time-lapse experiments, 6–12 spheroids for each experimental condition were collected, pooled and seeded on fibronectin-coated (final concentration: 10μg/ml) 6-well plate (one well for each condition). Spheroids were incubated for 1 hour at 37’C to allow their attachment, then spreading was monitored with Olympus ScanR or iXplore inverted microscope with 4X objective every 10 minutes for 48 hours (MCF10 DCIS.com) or every 20 minutes for 72 hours (Hacat), at 37°C in humidified atmosphere with 5% ${\text{CO}}_{2}$.

The contact area between the cell aggregates and the substrate was calculated at fixed time points (8 h, 16 h, 24 h) with Fiji. Its evolution over time was obtained via automatic segmentation of the time-lapse phase-contrast movies with custom MATLAB scripts. Only frames where the contact area had exceeded the initial spheroid projection of at least 20% were considered. The early stage spreading rate $\left(\frac{dA}{dt}\right)$ of each spheroid was then estimated by fitting the experimental curves with a linear fit of the kind $A(t)=\left(\frac{dA}{dt}\right)t+c$ over the first 24 hours. The initial spheroid size was estimated from the spheroid projected area $A_{0}$ in the first frame of the time-lapse acquisition as the equivalent radius $R_{0}=\sqrt{A_{0}/\pi }$.

Time-resolved cell velocity field was obtained via PIV analysis of time-lapse phase-contrast movies of spreading spheroids. PIV analysis was performed after masking the image portion outside the contact area to screen the background and calculate the velocity field only within the spreading monolayer. The same mask, obtained from contact area segmentation, was used for consecutive frames. The time interval between consecutive frames was 5 or 10 min and the interrogation windows were 8 or 16 pixels (26.1 or 20.8μm) on a side.

The radial velocity component in each grid point was computed by considering the scalar product $v_{r,i}=v_{i}\cdot {\overset{ˆ}{u}}_{r}$ where ${\overset{ˆ}{u}}_{r}$ is the unit vector pointing from the centroid of the contact area to the center of the i-th interrogation window. We then computed the radial velocity profiles $v_{r}(r,t)$ at a given time t, by performing an azimuthal average over the grid points at the same distance r from the centroid.

The radial velocity profiles were first averaged over 5 consecutive frames and then fitted with the analytical solution of $v_{r}(r)$ obtained from the model, which has the following form:

$$v_{r}\left(r\right)=(R(R_{1}^{2}-r^{2})\left(2AL_{c}^{2}+BR\right)I_{1}{\left(\frac{R}{L_{c}}\right)}^{2}-BR^{2}\left(R_{1}^{2}-r^{2}\right)I_{0}{\left(\frac{R}{L_{c}}\right)}^{2}-2AL_{c}^{2}rI_{1}\left(\frac{R}{L_{c}}\right)I_{1}\left(\frac{r}{L_{c}}\right)\;+L_{c}R\left(R_{1}^{2}-r^{2}\right)\left(B-2AR\right)I_{0}\left(\frac{R}{L_{c}}\right)I_{1}\left(\frac{R}{L_{c}}\right)+BL_{c}R_{1}I_{0}\left(\frac{R_{1}}{L_{c}}\right)I_{1}\left(\frac{R_{1}}{L_{c}}\right)\left(R^{2}+r^{2}\right)\;-BL_{c}r\left(R_{1}^{2}+R_{2}^{2}\right)I_{0}\left(\frac{r}{L_{c}}\right)I_{1}\left(\frac{r}{L_{c}}\right)\;+2AL_{c}^{2}R_{1}I_{1}\left(\frac{R_{1}}{L_{c}}\right)I_{1}\left(\frac{R}{L_{c}}\right)\left(R^{2}+r^{2}\right))/2\;r\;I_{1}{\left(\frac{R}{L_{c}}\right)}^{2}\left(R_{1}+R^{2}\right)$$

where $A=T_{0}h/\eta ,B=-\zeta /\eta ,L_{c}$ is the nematic length, $R_{1}$ is the position at which the radial velocity vanishes (representing the size of the solid core), R is the position of the spreading front, and $I_{n}$ is the modified Bessel function of the first kind and n-th order.

The analytical solution was fitted only up to the radial position corresponding to 1.07% $r_{p}$, where $r_{p}$ is the position of the maximum of the experimental velocity profile, to reduce artefacts coming from boundary jags. For the fit to converge, R was fixed to the boundary value, while $A,B,R_{1}$, and $L_{c}$ were kept as free fit parameters.

### Mammary Epithelial Cells purification & spheroid formation

Mammary Epithelial Cells (MECs) were obtained by mammary glands of C57Bl/6J female mice (7–10 weeks old), either *wild type* or KO for IRSp53^15^. All mice have been maintained in a controlled environment, at 18–23 °C, 40–60% humidity and with 12-h dark/12-h light cycles, in a certified animal facility under the control of the institutional organism for animal welfare and ethical approach to animals in experimental procedures (Cogentech OPBA). All animal studies were conducted with the approval of Italian Minister of Health (1192/2020-PR) and were performed in accordance with the Italian law (D.lgs. 26/2014), which enforces Dir. 2010/63/EU (Directive 2010/63/EU of the European Parliament and of the Council of 22 September 2010 on the protection of animals used for scientific purposes).

Animals were euthanized with a high concentration of ${\text{CO}}_{2}$ and mammary glands were excised *post mortem*.

MECs were purified as previously described^56^. Briefly, after excision mammary glands were immediately transferred into 50 ml tubes with 5 ml of recovery medium (DMEM/F-12 + GlutaMAX, 25mM HEPES, Pen/Strep) and subjected to overnight incubation at 37°C into 50 ml tubes with 5 ml of digestion medium (DMEM/F-12 + GlutaMAX, 25mM HEPES, Pen/Strep, 150UI/ml Collagenase Type 3, 20μl/ml Liberase TM). The day after (day 2), tissues were mechanically dissociated by vigorous pipetting and cell suspension was transferred to a new 50 ml tube with additional 25 ml of recovery medium. After a centrifugation of 1500 rpm for 5 minutes at room temperature, the interface was removed and the cellular pellet was resuspended in 10 ml of 0,25% Trypsin-EDTA, for a digestion step of 20 minutes. Trypsin was then inactivated by adding 20 ml of stop medium (DMEM/F-12 + GlutaMAX, 25mM HEPES, Pen/Strep, supplemented with 10% FBS) and a second centrifugation 1500 rpm for 5 minutes at room temperature was performed. After discarding the supernatant, the cellular pellet was resuspended in MEGM medium (CC-3150, Lonza), seeded in a 6-well and subjected to overnight incubation at 37°C. The successive day (day 3), cellular contaminants (such as fibroblasts and other undesired cell types) were removed from MEC culture by performing a short incubation with 0.5 ml of 0,1% Trypsin-EDTA for 2 minutes. After trypsin inactivation with stop medium, the surnatant was removed and epithelial cells were cultured at 37°C in MEGM medium for 2 additional days.

MEC spheroids were obtained by seeding 5*10^3^ cells in 200μl of complete medium in 96-well Clear Round Bottom Ultra-Low Attachment Microplate (7007 – Corning) and centrifuging them for 5 minutes at 1200 rpm, to allow the gathering of cells at central area of each well. After 5 days, compact roundish spheroids are formed (one for each well). Wetting experiments were performed as previously described for MCF10DCIS.com spheroids.

### Traction force microscopy & analysis

SCR and IRSp53_KD spheroids were obtained by seeding 3*10^5^ cells in 2 ml of complete medium with doxycycline 1μg/ml in AggreWellTM400 24-well microplate (34411 – StemCell), according to manifacturer’s instructions (one well for each condition). After 16 hours, around 1200 spheroids composed by ~ 250 cells/each are obtained.

Traction force microscopy substrates were prepared as previously described^57^. Briefly, a 15-kPa soft PDMS mixture was prepared by mixing DOWSIL CY 52–276 A and B components in 1:1 ratio (w/w). The mixture was spin-coated onto glass coverslips (spin acceleration 250 rpm/s, 500 rpm for 10s, then 750 rpm for 40 s) and polymerized overnight at 70 °C. PDMS layers were then activated with a 1:10 (v/v) solution of 3-aminopropyltriethoxysilane (Sigma-Aldrich) diluted in 100% lab-grade ethanol for 10 min at RT, washed thrice with 100% ethanol and dried at 70 °C for 10 min. Substrates were functionalized with a 2:1000 (v/v) solution of fluorescent beads (FluoSpheres carboxylate-modified microspheres, 0.2μm, Thermo Fisher Scientific) in MilliQ water was sonicated for 5 min, filtered through 0.45μm mesh and incubated with the activated soft PDMS surfaces for 10 min at RT. Substrates were then washed thrice with MilliQ water and dried at 70 °C for 10 min. TFM surfaces were kept at RT in the dark no longer than 2 weeks.

Soft PDMS substrates were coated with fibronectin (final concentration: 7μg/ml) and spheroids were seeded on the gels after harvesting step. Z-stacks of fluorescent bead images and brightfield images were acquired every 10 min using a Nikon TI2 microscope (as option equipped with CSU-W1 Yokogawa Spinning Disk unit) with a 40× NIKON Plan-Apochromat air objective (NA = 0.95, WD=250μm) and environment control $\left(37{}^{\circ}\text{C},5\%{\text{CO}}_{2}\right)$. At the end of the time-lapse imaging, spheroids were detached from the substrate using 10 % (w/w) sodium dodecyl sulfate (SDS) solution in order to acquire the relaxed position of the beads.

At each timepoint, the fluorescent beads within the gel were compared to a reference image obtained after cell detachment at the end of the experiment. Displacements were computed from bead trajectories obtained with single particle tracking using TrackMate^58,59^.

The instantaneous traction field was obtained from the displacement field using a custom MATLAB script implementing the Fourier transform traction cytometry (FTTC) scheme^60^, with 0^th^ order Tikhonov regularization^61^.

Radial traction force components were computed and then azimuthally averaged over the points at the same distance r from the centroid of the contact area to get the radial traction profiles $T_{r}(r)$ at a given time t.

At each time t, we fit the experimentally measured radial traction force profiles with the equation $T_{r}(r)=-T_{0}I_{1}\left(\frac{r}{L_{c}}\right)/I_{1}\left(\frac{R}{L_{c}}\right)$ predicted by the model^9^, to estimate the maximal traction stress $T_{0}(t)$ and the nematic length $L_{c}(t)$.

The theoretical prediction was fitted only up to the radial position of the maximum of the experimental traction force profile, which sets the monolayer radius R(t), discarding the outer region where $T_{r}(r)$ drops because of poorly attached protrusions or long-range propagation of deformations in the elastic substrate^9^.

### Spheroid deformation in microfluidic device

SCR and IRSp53_KD spheroids were obtained by seeding 1.2*10^5^ cells in 2 ml of complete medium with doxycycline 1μg/ml in AggreWellTM400 24-well microplate (34411 - StemCell), according to manifacturer’s instructions (one well for each condition). After 16 hours, around 1200 spheroids composed by ~ 100 cells/each are obtained.

Microfluidic devices were designed using AutoCAD software, and silicon wafer masters were produced in a clean room environment (PoliFAB, Politecnico di Milano). The final devices were fabricated using molds and elastomer polydimethylsiloxane (PDMS, Dow Corning SYLGARD 184 Silicone Elastomer Kit 1.1 KG KIT, Dow Corporate). To fabricate the device, the cured replicas were peeled off the mold, punched, and plasma bonded to thickness no. 1 glass coverslips (Prestige) that were pre-coated with a thin layer of PDMS. The bonded chips were kept at 80°C for 20 min to secure a robust bonding. Afterwards, the silicon tubing was attached to the inlet and outlet of the devices. Before use, the devices were conditioned with 5% Pluronic F68 (P1300 – Sigma-Aldrich) in PBS for 24 hours to reduce the non-specific adsorption.

Microfluidic channels were incubated with an anti-adhesion coating solution (07010 – Stem Cell) for 15 minutes, followed by a wash with media. Spheroids were introduced into the devices (for full technical details, please refer to B.G. and G.S., manuscript in preparation) at a constant flow rate of 3ml/h: approximately 120 spheroids were introduced in 200μl of media containing 25 mM Hepes.

Deforming spheroids were tracked using a Thunder Leica microscope and a Camera (Photometrics Prime BSI Express sCMOS) camera. Videos were created using a 5X objective using the brightfield channel and obtaining 4–5 ms time step sizes. Deformation passages were recorded manually by recording the inlet and outlet frame at which a spheroid passes through a constriction channel and multiplying by the time step.

### Spheroid fusion

For fusion experiments, spheroids were obtained by seeding 500, 2500 or 5000 cells in 96-well Clear Round Bottom Ultra-Low Attachment Microplate as previously described. Once formed, two spheroids of the same size were collected and pooled in the same Round Bottom well, in close proximity. EDTA was added at the final concentration of 0.3 mM, while seeding cells in Ultra-Low Attachment Microplate to get spheroids. Dynamics of coalescence was monitored by acquiring images with EVOS stereo-microscope using 4X objective after 1, 8, 24, 48 and 72 hours. We followed the growth of the angle radius r(t) of the neck between the coalescing spheroids. By fitting a simple exponential function of the kind $a\left(1-e^{-\Gamma t}\right)$ to the time evolution of the normalized variable $(r/R{)}^{2}$, we estimated the initial fusion rate $\Gamma ≃\sigma /\eta R_{0}$ and the amplitude term $a=(r/R{)}_{\infty }^{2}$, which is expected to tend to 1 for complete fusion.

### 3D sorting in spheroids

Spheroids were obtained by mixing SCR (stably expressing EGFP-E.cadherin) and IRSp53_KD (stably expressing LifeAct-mCherry) cells in a 1:1 ratio and seeding 5*10^3^ cells in 200μl of complete medium with or without doxycycline 1μg/ml in 96-well Clear Round Bottom Ultra-Low Attachment Microplate (7007 – Corning), as previously described.

After 48 hours from cell seeding, spheroids were imaged with EVOS stereo-microscope with a 10X objective. Sorting patterns of SCR_GFP-Ecadherin and IRSp53-KD_LifeAct-mCherry cells were quantified by manually contouring with Fiji the areas respectively occupied within each spheroid and calculating their ratio (SCR-GFP/IRSp53_KD-mCherry).

### Tipless AFM of single cell cortical tension

Tipless AFM-base force spectroscopy measurements on single cells were conducted on 35 mm glass bottom dishes (FluoroDish) coated with a thin layer of 0.01% Poly-L-Lysine (P4707 – Sigma Aldrich). Cells were trypsinized and resuspended in complete medium until ready to use on the AFM. Tipless cantilevers (HQ:CSC38/tipless/Cr-Au; MikroMasch) were calibrated in 1x PBS and then equilibrated to 37°C. Next, cells were seeded on Poly-L-Lysine coated dishes and transferred to the AFM stage. Tipless deformations were conducted using a 3μm ramp size with 1.5μm/s speed, performing deformations no greater than 1000nm in Z-length. Five consecutive force curves were collected for each cell, allowing the cell to relax for 15–20 seconds in between each force curve. In addition to analyzing untreated cells, the effects of actomyosin inhibition and activation were tested with blebbistatin treatment (50μM, 20 minutes pre-treatment) and CN-03 treatment (10μg/mL, 4 hours pre-treatment), respectively. Tipless analysis was conducted using previously described methodologies analyzing the first 500nm of the contact regime for each force curve^44^.

### Tipless AFM of single cell cortical tension within spheroids

SCR and IRSp53_KD spheroids were obtained by seeding 6*10^5^ cells in 2 ml of complete medium with doxycycline 1μg/ml in AggreWellTM400 24-well microplate (34411 – StemCell), according to manifacturer’s instructions (one well for each condition). After 16 hours, around 1200 spheroids composed by ~ 500 cells/each are obtained (diameter below 150 um).

To adhere spheroids, 35mm glass bottom dishes (FluoroDish) were coated using CellTak tissue adhesive (354240 – Corning). As with single cells, tipless cantilevers (HQ:CSC38/tipless/Cr-Au, MikroMasch) were calibrated in 1x PBS, then spheroids were gently mixed, deposited onto CellTak-coated dishes and transferred to the AFM stage. Similar force parameters to single cell analysis were used to deform surface-level cells on the top of well-adhered spheroids. Once again, the first 500nm of each force curve was used to analyze tension values for each set of data.

### FLIM to measure membrane tension

Cells seeded as sparse islands or confluent monolayers were incubated in complete media supplemented with 1μm of the Flipper-TR (Spirochrome/TebuBio SC020) for at least two hours before the experiment.

Fluorescence lifetime imaging microscopy experiments were performed on a Leica TCS SP8 laser-scanning confocal module mounted on a Leica DMi 8 inverted microscope, equipped with a time-correlated single-photon counting module (TCSPC, PicoQuant), on-stage environmental chamber (Okolab), and controlled by the software Leica Application Suite X (ver. 3.5.2.18963).

For image acquisition, a HC PL APO CS2 40 ×/1.30 oil immersion objective was used. Samples were excited with a pulsed White Light Laser operating at 20 MHz frequency, 1–5% power and the excitation wavelength set at 488 nm. Emission signal was collected through a 550–650 nm bandpass filter using Hybrid detectors (HyDs) in photon counting mode, pinhole set to 2 AU and 200 Hz scanning speed to maximize photon detection. A signal intensity of at least 10^4^ A.U. and an average of 300 photon/pixel was collected for each image.

Lifetime analysis was performed on the software SymPhoTime64 (Picoquant). Specifically, spatial resolution was sacrificed in favor of maximum fitting likelihood and 512×512 raw images were binned 2x (256×256). The Two-Exponential tail-fit method was applied to extract fluorescent lifetimes and excludes the potential contribution of the sample excitation during laser pulses. Given the negligible contribution of $\tau_{1}$ compared to $\tau_{2}$ of the decay curve, average lifetime was calculated for whole images, setting the pixel with >20–30 event counts to NaN. For islands analysis, events count images were used to create arbitrary binary mask of cell-cell junctions, to avoid contribution from non-junctional portions of the cells to lifetime calculations. Mean lifetime values for each thresholded image were obtained using Fiji.

### Blebbing analysis

SCR and IRSp53_KD cells were detached by trypsinization and resuspended by gentle pipetting for 5 minutes. Blebs formation and re-absorption over time was monitored by DeltaVision microscope with 40X oil objective every 10 seconds for 5 minutes. Blebbing dynamics was quantified by segmentation of cells with a custom Fiji plugin^62^. After choosing the object to measure for each cell, the plugin applies a WEKA custom model^63^ to identify the objects and extrapolates the area variation for each frame.

### Nanomechanical AFM mapping of MCF10DCIS.com monolayer

SCR and IRSp53_KD cells were seeded in confluent conditions on 35mm glass bottom dishes (FluoroDish FD35–100 - World Precision Instruments), in complete medium with doxycycline 1μg/ml. The day after, monolayers were rinsed with PBS 1x several times and taken to the NanoWizard 4XP AFM system (Bruker). After calibrating a PFQNM-LC-V2 live cell probe (Bruker) and letting it equilibrate to 37°C, the cells were placed on the AFM stage to image. Cells were scanned using Quantitative Imaging mode to generate high spatial resolution maps to quantify Young’s modulus using a modified Hertz contact model for paraboloidal tips^64^. Scans were taken at 256*256 resolution with a 1nN force setpoint, 1500nm Z range, and a 100μm/s ramp speed over a $50*50\mu {\text{m}}^{2}$ area.

### Laser ablation on junctions & apical cortex

SCR and IRSp53_KD cells (stably expressing EGFP-CAAX as membrane reporter) or AFD_KD cells (stably expressing mCherry-CAAX) were seeded in confluent conditions (at jamming density) onto Nunc Glass Base dish (diameter of 27 mm) in complete medium with doxycycline 1μg/ml.

After 48–72 hours, monolayers were subjected to targeted laser ablation. Experiments were performed using a Leica Stellaris DIVE combined with a femtosecond pulsed Spectra Physics Laser capable of priming two-photon excitation. The ablation was performed using a 750 nm wavelength. Imaging was performed with single photon excitation in a regular confocal fashion. The objective used was a HC PL APO 63x/1,2 W UVIS CS2. For laser ablation on junctions, the distance between vertices (vertex separation) defining the ablated contact was measured as a fraction of time. Distance values were subtracted from the initial contact length. The values were then calculated as a function of time, and initial recoil values for each contact were obtained by nonlinear regression of the data to the following equation: f(t)= (initial recoil/k) (1–e-kt)^7,65^. For laser ablation of apical cortex, changes in cellular area (measured by CAAX signal as proxy for cell perimeter) were quantified as a fraction of time using Fiji software^29^.

### Correlative light and electron microscopy (CLEM)

Electron microscopic examination and correlative light-electron microscopy (CLEM) were performed as previously described^21,66,67^. Briefly, MCF10 DCIS.com SCR and IRSp53_KD cells (stably expressing EGFP-E-cadherin as junctional reporter) were trypsinized to a single cell suspension at 4*10^4^ cells/ml in complete medium containing 2% Matrigel (BD Biosciences). Cell-medium-Matrigel suspensions (350μl) were plated into 35 mm dishes with gridded coverslips (P35G-1.5–14-CGRD - MatTek Corporation), pre-coated with 20μl Matrigel (10mg/ml). 2% Matrigel complete medium was added to 2 ml of complete medium, with doxycycline 1μg/ml. After 72–96 hours, obtained spheroids were stained with NucBlue^™^ Live ReadyProbes^™^ and identified on grids by confocal microscopy. The fluorescent images acquired for the Correlative Light Electron microscopy data were acquired on a Leica SP8-DLS confocal microscope, using either a HC PL Fluotar 20x/0.5 objective.

#### Electron microscopy:

A brief description of each process is presented below.

##### Embedding:

spheroids grown on Mat Tek glass bottom dishes were fixed with 2.5% paraformaldehyde and 2,5% glutaraldehyde (EMS, USA) mixture in 0.2M sodium cacodylate pH 7.2 for 2h at RT, followed by 3 washes in 0.2M sodium cacodylate pH 7.2 at RT. Then, spheroids were incubated in 1:1 mixture of 2% osmium tetraoxide and 3% potassium ferrocyanide for 1h at RT followed by 3 times rinsing in cacodylate buffer. Afterwards, samples were sequentially treated with 0.3% Thiocarbohydrazide in 0.2M cacodylate buffer for 15 min and 1% OsO4 in 0.2M cacodylate buffer (pH 6,9) for 30 min. Samples were then rinsed with 0.1M sodium cacodylate (pH 6.9) buffer until all traces of the yellow osmium fixative have been removed, washed in deionized water, treated with 1% uranyl acetate in water for 1 h and washed in water again. Finally, samples were subjected to dehydration in ethanol and embedded in Epoxy resin at RT and polymerized for at least 72 h in a 60 °C oven^66^.

##### Sectioning.

As described above, the spheroid of interest was selected during the optical sectioning and the Z-stacking using confocal microscope. During Z-stacking the distance between the bottom and the surface of the spheroid was estimated. Embedded samples were then sectioned with diamond knife (Diatome, Switzerland) using Leica EM UC7 ultramicrotome. For the trimming and advance to ROI we used the Diatome diamond trimming blades (trimtool 45, and histo) (Diatome, Switzerland). To avoid mistakes we took into consideration the possible “shrinkage” of the matrix during its dehydration and embedding into Epon. Thus, when only 3 microns were left before the beginning of the cell surface, we replaced the trimming histo-knife with the Ultra 35 knife (Diatome, Switzerland), and then cut two 200-nm sections and then a small series of 70 nm sections. Sections were analyzed with a Tecnai 20 High Voltage EM (FEI, now Thermo Fisher Scientific; The Netherlands) operating at 200 kV.

### Focused Ion Beam-Scanning electron microscopy (FIB-SEM)

Samples were mounted on SEM support stubs (Agar Scientific). The resin blocks were glued to the stub and surrounded by silver paint leaving out the trimmed pyramid with sectioned area containing exposed spheroids. The mounted samples were sputter-coated with iridium using 10mA sputter current for 120 sec (EMITECH K575X) and transferred into Crossbeam 550 (ZEISS Microscopy GmbH, Oberkochen, Germany). The cell contours within each spheroid were visualized by SEM imaging using SE2 detectorat EHT 5 kV and were correlated with corresponding FIB images for targeting the volume of interest. FIB-SEM volume imaging was performed using Atlas3D package (Zeiss Microscopy, Oberkochen, Germany). On top of the sample surface a protective pad was prepared with specific Atlas3D tracking marks used to correct for sample drift with respect to the SEM imaging or FIB Milling on-the-fly during the experiment. This allows to determine the individual slice thickness and to maintain the target slice thickness throughout the process which is required for the thinnest slices. For the control sample (SCR) the lateral pixel size was 5 nm and the FIB slice thickness was 10 nm. The FIB slices were milled using 30kV:700 pA FIB probe and imaged with 500 pA SEM probe current at 1 kV acceleration potential using the Energy selected Back-scatter (EsB) detector (EsB Grid set to 737 V), dwell time 3μsec and line average (N=3). 1495 slices were used of 1770 acquired slices in 45h 25min. The ROI imaged was 2560*3150 pixel resulting in a volume of $12,75*15.75*14,95\mu {\text{m}}^{3}$. For the KD sample a first data set of 854 slices (750 used) was collected in about 20 h 30 min with the same parameter but EsB grid set to 727 V and a ROI of 2665*2810 resulting in a volume of $13.33*14.05*7.5\;\mu {\text{m}}^{3}$. A second data set consisting of 2064 slices (1839 used) was acquired in 51 h and 13 min using a lateral pixel size of 2.5nm and a slice thickness of 5nm. The FIB probe current was 30kV:300 pA and the SEM Parameter were equal besides EsB Grid set to 650 V and line average N=2. The imaged ROI of 2100*5550 pixel was resulting in an acquired volume of $5.25*8.33*9.2\mu {\text{m}}^{3}$.

Alignment and image export by Atlas5 package.

Image processing using Dragon fly.

### Immunofluorescence on MCF10 DCIS.com monolayers

SCR and IRSp53_KD cells were seeded in confluent conditions (at jamming density) in μ-Slide 8 well ibiTreat chambers (Ibidi – 80826) in complete medium with doxycycline 1μg/ml. After 16 hours, monolayers were fixed with 4% PFA for 20 minutes at room temperature. Cells were then permeabilized and blocked with PBS 0.1% Triton-X100, 0.2% BSA for 20 minutes at room temperature. Incubation with primary antibody solution (1% BSA in PBS 1x, for dilutions please refer to the Reagents List) was performed overnight at 4’C in a wet chamber protected from dark. After 3 washes of 5 minutes/each with PBS 1x, incubation with secondary antibody solution (PBS 1x, for dilutions please refer to the Reagents List) was performed for 1.5 hours at room temperature. After 3 washes of 5 minutes/each with PBS, nuclei were stained with Dapi (1:1000 in PBS 1x) and samples were stored in PBS at 4’C till acquisition.

Samples were imaged by Leica SP8 DLS microscope (used lasers: 405, 488, 561 & 647 nm) with a 63X (1.4 correction) oil objective. Confocal sections on z axis were acquired with 0.5μm step-size and resliced on y axis using ImageJ, to get maximal projections of actin staining (TRITC-phalloidin). A Leica algorithm allowing for computational clearing (Thunder) was also used to image phallodin-positive intercellular spaces in MCF10 DCIS.com monolayer.

### E-cadherin and β1-integrin surface quantification

For FACS-based quantification of cell surface E-Cadherin (ECAD), active β1-integrin, and total β1-integrin, adherent SCR and IRSp53_KD cells were labeled *in vivo* at 4°C with anti-ECAD (HECD-1) at 2μg/ml, anti-active β1-integrin (9EG7) at 10μg/ml, or anti-total β1-integrin (TS2/16) at 1μg/ml for 1 hour, followed by incubation with anti-mouse (for ECAD and total β1-integrin) or anti-rat (for active β1-integrin) Alexa-488 conjugated secondary antibody for 30 minutes at 4°C. Cells were then washed with ice-cold PBS 1x, detached using 0.25% trypsin, subsequently fixed with 2% formaldehyde in PBS on ice for 15 min.

After fixation, cells were washed and resuspended in PBS supplemented with 2 mM EDTA and further processed for FACS analysis using a BD FacsCelesta. Analysis was performed using FlowJo software (LLC). Cell doublets and clumps were excluded by analyzing the side scatter area (SSC) versus the height peak parameter. Cells with the correct morphology were selected using the forward scatter area (FSC) versus SSC area parameters. The mean fluorescence intensity of surface proteins in SCR and IRSp53_KD samples was extrapolated, subtracted from background (i.e., the mean fluorescence intensity from cells labeled with the secondary antibody alone) and normalized to SCR sample.

### Cell spreading for focal adhesions analysis

SCR and IRSp53_KD cells were detached by trypsinization, seeded onto fibronectin-coated (10μg/ml) glass bottom dishes in sparse condition and fixed with 4% PFA 1h or 6h after seeding. Cells were subjected to immunofluorescence analysis, with a-vinculin antibody (V9131 - Sigma-Aldrich) to identify focal adhesions, phalloidin for F-actin and DAPI. TIRF imaging of focal adhesions was performed on a Leica TIRF microscope, using a HC PL APO 63x/1,47 OIL CORR TIRF. To segment cells and see the nucleus in the same image, we combined imaging of DAPI using epi-illumination.

CellProfiler 4^68^ was used to measure focal adhesions number and area. The pipeline identifies the nuclei using the IdentifyPrimaryObjects module with two classes parameters after Otsu’s threshold method on the DAPI channel. Then, the cell boundaries are recognized using the IdentifySecondaryObjects module with propagation method on the phallodin channel, using the nuclei identified as primary objects. At last, the pipeline applies the EnhanceOrSuppressFeatures module on vinculin channel and segments the Focal Adhesion using, on the enhanced image, the IdentifyPrimaryObjects module with three classes parameters after Otsu’s threshold method. RelateObjects and MeasureObjectSizeShape modules were used to count and measure the focal adhesion for each cell.

### Standard Cell lysis for WB analysis

After washing with PBS 1x, cells were lysed in cell lysis buffer (Hepes pH 7.5 50mM, NaCl 150 mM, glycerol 1%, Triton X-100 1%, MgCl_2_ 1.5mM, EGTA 5mM, 1 mM DTT, EDTA-Free, PIC) directly on the plates using a cell-scraper. Lysates, transferred in 1.5 ml tubes, were incubated on ice for 10 minutes and spun at 13200 rpm for 10 min at 4°C. The supernatant was transferred into a new 1.5 ml tube and protein concentration was measured by the Bradford assay (Biorad), according to manufacturer’s instructions.

### SDS polyacrylamide gel electrophoresis (SDS PAGE)

Pre-casted acrylamide gels (BIORAD or Thermo Fisher Scientific) were employed for resolution of proteins.

### Western blot analysis

Desired amounts of proteins were loaded onto pre-casted acrylamide gels (BIORAD or Thermo Fisher Scientific). Proteins were transferred in Western transfer tanks (Biorad) to nitrocellulose (Schleicher and Schuell) in Western Transfer buffer 1x (diluted in 20% methanol) at constant Voltage (100V for 1 hour or 30V overnight). PonceauS staining was used to reveal roughly the amounts of proteins transferred on the filters. Filters were blocked 1 hour (or overnight) in 5% milk or 5% BSA in TBS 0.1% Tween (TBS-T).

After blocking, filters were incubated with the primary antibody (for dilutions please refer to the Reagents List), diluted in TBS-T 5% milk, for one hour at room temperature, or overnight at 4°C, followed by three washes of five minutes each in TBS-T and then incubated with the appropriate peroxidase-conjugated secondary antibody (for dilutions please refer to the Reagents List) diluted in TBS-T for 1 hour. After the incubation with the secondary antibody, the filter was washed three times in TBS-T and the bound secondary antibody was revealed using the ECL (Enhanced Chemiluminescence) method (Amersham). Images were acquired with either ChemiDoc Imagers (BioRad) or Invitrogen^™^ iBright^™^ Imagers (Thermo Fisher Scientifics).

All uncropped blots can be found in the source data file.

### Co-immunoprecipitation assay

Lysates prepared in IP buffer (50mM Hepes pH7.5, 150mM NaCl, 10mM MgCl2, 2mM EDTA, 10mM NaPyr, 50mM NaF, 10mM NaVan, 2mM PMSF, 1mM DTT, PIC) were incubated in the presence of the anti-FLAG M2 antibody (SIGMA) and Protein G Sepharose resin (SIGMA) for two cycles of 1.5 hour each at 4°C with rocking. Immunoprecipitates were washed 3 times in IP buffer. After washing, beads were resuspended in 1:1 volume of 2x SDS-PAGE Sample Buffer, boiled for 10 min at 95°C, centrifuged for 1 minute and then loaded onto polyacrylamide gels.

### *In vitro* binding

Cell lysates from HEK T293 cells transfected with GFP-Afadin (lysis buffer: 50mM Hepes pH7.5, 150mM NaCl, 10mM MgCl2, 2mM EDTA, 10mM NaPyr, 50mM NaF, 10mM NaVan, 2mM PMSF, 1mM DTT, PIC) were incubated with equal amount (0.5μM) of GST-IRSp53 fragments or GST as control, 1 hour and 30 minutes at 4 °C. Reactions were washed 3 times in lysis buffer. After washing, beads were resuspended in 1:1 volume of 2x SDS-PAGE Sample Buffer, boiled for 10 min at 95°C, centrifuged for 1 minute and then loaded onto polyacrylamide gels.

### GST-fusion proteins production

All the GST fusion proteins used were product in bacteria using *E. coli* BL21 Rosetta (DE3) competent cells transformed with the pGEX6P1 in which the desired construct had been cloned.

### Bacterial culture

*E. coli* BL21 Rosetta (DE3) cells picked from individual colonies, transformed with the indicated GST-fusion, were used to inoculate 200 mL of LB medium (containing ampicillin at 50 mg/mL) and were grown overnight at 37°C. Between 10 and 100 mL of the overnight culture was diluted in 1 litre of LB and was grown at 37°C (240 rpm shaking) till it reached approximately OD=0.4–0.6. IPTG (1mM) was then added used to induce the protein production. After the induction cells were pelleted down at 6000 rpm for 15 minutes at 4°C and pellets were used immediately or conserved at −80°C after washing in PBS 1X.

### GST-fusion protein

Pellets were suspended in GST-lysis buffer (15mL for 1L culture). Samples were sonicated 3 times for 30 seconds/each on ice and were pelleted down at 13200 rpm for 30 minutes at 4°C using a JA 20 Beckman rotor or at 40000 rpm for 45 minutes at 4°C using a 55.2 Ti Beckman rotor. 1 mL of glutathione-sepharose beads (Amersham), previously washed 3 times with GST-lysis buffer, was added to the supernatant and samples were incubated 1–2 hour at 4°C while rocking. Beads were then washed 3 times (with 5 minutes of incubation at 4°C each) in the GST lysis solution. GST-proteins were resuspendend 50% slurry in the GST-lysis solution. The quantification was achieved in an SDS PAGE gel using a titration curve with BSA.

### GST-lysis buffer

PBS, 0.5 mM EDTA, 10% Glycerol, Protease inhibitor cocktail (Roche, Basel, Switzerland) (freshly added), 1mM DTT (freshly added).

### Statistical analysis

All data are presented as scatter plots with bars and the mean of independent biological replicates ± SD. The number of experiments as well as the number of samples analyzed is specified for each experiment and reported in the figure legends. The p values, when statistically relevant, are specified in each graph.

P values were derived as follow:

1. Unpaired t-test with Welch’s correction (0.05) - Fig. 1C, 2A (dA/dt), 2D–G, 4A, 5A, 6D, S1G–H, S1I (dA/dt-5000), S1L–N, S2A (Number of FA/cell-6h and FA area/cell-1h), S2B, S3A–B.
2. Mann-Whitney test (0.05) - Fig.2 I–L, 3A, 4B–C, 5B–D, 6A, S1A, S1I (dA/dt-250, 1000), S1O, S2A (Number of FA/cell-1h and FA area/cell-6h).
3. Brown-Forsythe and Welch ANOVA tests (0.05) - Fig.1B.
4. 2way ANOVA (0.05) - Fig.1D, 2A (Area), 3C, 4D–F, 6C, 7A, 7D, S1D–E, S2C–F, S3C–E.

## Supplementary Material

1
