# Supplementary material for: Biophysical and Molecular mechanisms that control active wetting and tissue fluidification in epithelial tissues
Source: Res Sq. 2025 Mar 3:rs.3.rs-6008502. Preprint. [Version 1] doi: 10.21203/rs.3.rs-6008502/v1 (PMC11908352; doi:10.21203/rs.3.rs-6008502/v1)
Supplement: 1 [file NIHPPRS6008502V1-supplement-1.pdf]

Supplementary Information

- **Supplementary Figure S1**
- **Supplementary Figure S2**
- **Supplementary Figure S3**

- **Supplementary Movies Legends**

Supplementary Figure S1

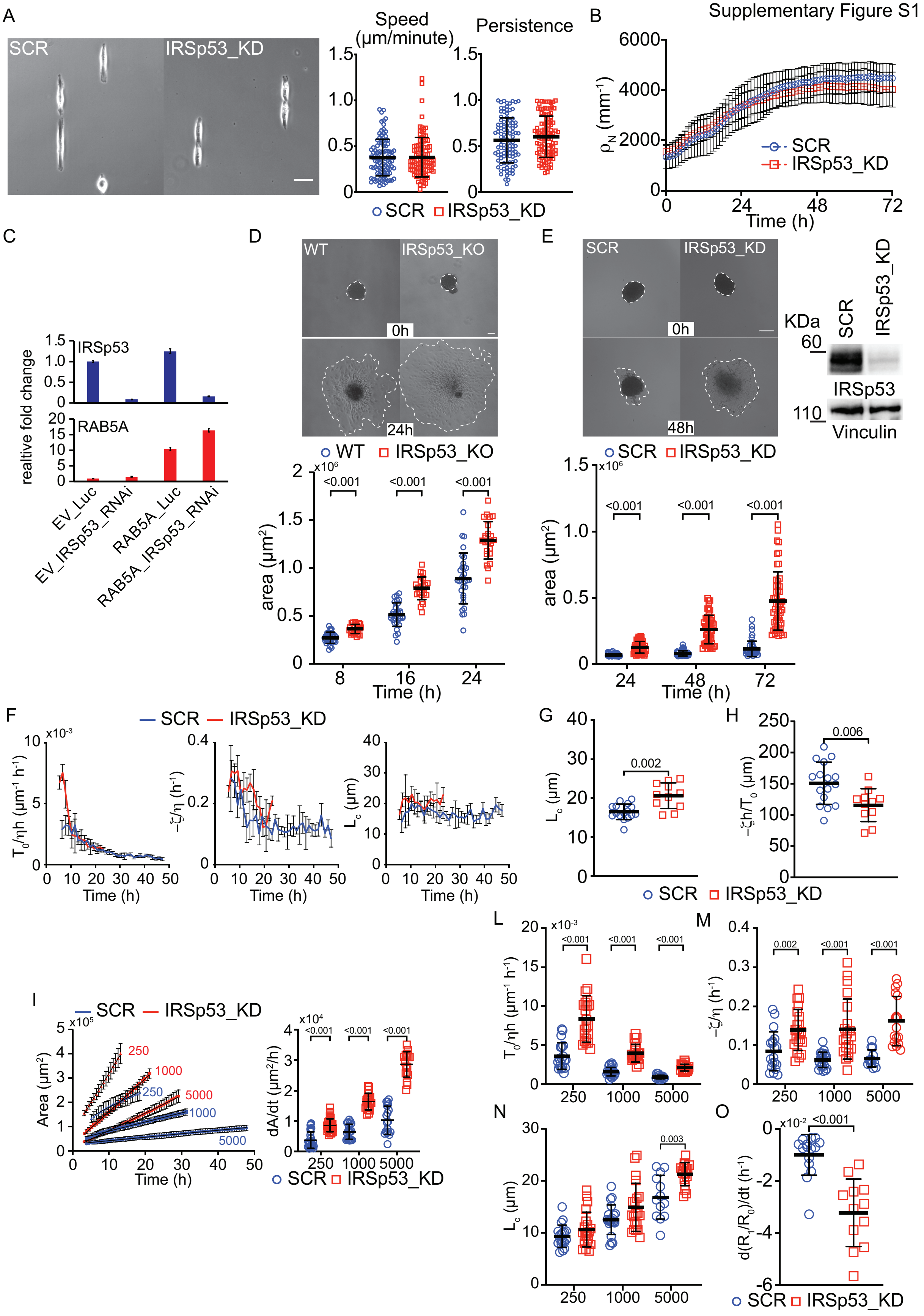

Supplementary Figure S1. **A**. IRSp53 loss does not affect single cell migration. Doxycycline-treated MCF10 DCIS.COM control (SCR) or IRSp53 silenced (IRSp53_KD) cells were seeded on fibronectin (10µg/ml) coated linear patterns (10µm width) (please see Material and Methods). 16h after seeding cells were imaged by time lapse microscopy (Supplementary Movie S2). Left, still images are shown. Scale bar, 50µm. Right graphs, analysis of the mean instantaneous speed and mean persistence was performed with a developed C++ software coupled with R [55]. Data are mean ± SD (n=100 cells for each condition from 3 independent experiments). **B**. Nuclear density analysis over time of doxycycline-treated SCR-H2B-GFP and IRSp53_KD-H2B-mCherry MCF10 DCIS.COM cells seeded at jamming density and monitored by time-lapse microscopy (Fig. 1C and Supplementary movie S4). Data are mean ± SD (n=16 fields from 2 independent experiments). **C**. Expression of IRSp53 and RAB5A (related to Fig. 1D). mRNA levels were verified by qRT-PCR (mRNA fold increase relative to the levels of control cells after normalizing for GAPDH mRNA levels). **D**. Mammary glands epithelial cells derived from WT (WT) or IRSp53 null (IRSp53_KO) mice were cultured in ultra-low adhesion condition to form spheroids. Spheroids were then seeded on fibronectin coated 6 well plates and spreading monitored by time-lapse microscopy (Supplementary Movie S7). Representative still images at the indicated time points are shown. Scale bar, 100µm. Lower graph, spreading area at the indicated time points was manually quantified using ImageJ. Data are mean ± SD (n=31 WT, 25 IRSp53_KO spheroids from 3 independent experiments). **E**. Doxycycline-treated HaCat control (SCR) or IRSp53 silenced (IRSp53_KD) cells were cultured in ultra-low adhesion condition to form spheroids. Spheroids were then seeded on fibronectin coated 6 well plates and spreading monitored by time-lapse microscopy (Supplementary Movie S8). Upper left, representative still images at the indicated time points are shown. Scale bar, 200µm. Lower left graph, spreading area at the indicated time points was manually quantified using ImageJ. Data are mean ± SD (n=46 SCR, 51 IRSp53_KD spheroids from 3 independent experiments). Right, WB analysis, with the indicated antibodies, was performed to detect IRSp53 levels. **F-H**. Spreading parameters obtained from image segmentation and PIV analysis SCR and IRSp53_KD spreading spheroids (related to Fig. 2 B-G). F. From left to right, time evolution of A, B and L_c. G. Quantification of the nematic length L_c. Data are means ± SD (n=16 SCR, 11 IRSp53_KD spheroids from 3 independent experiments). H. Ratio B/A=$-\zeta h/T_0$ showing a difference between SCR and IRSp53-KD spheroids, suggesting a reduction in tissue contractility upon IRSp53 knockdown. Data are mean ± SD (n=16 SCR, 11 IRSp53_KD spheroids from 3 independent experiments) **I.** Left, the spreading area of spheroids of different size, quantified with semi-automatic image segmentation with MATLAB to follow its evolution over time. The solid black lines represent linear fits to the data of each condition. Right, rates dA/dt quantified for single samples. Data are mean ± SD (n=29 SCR-250, 34 IRSp53_KD-250, 21 SCR-1000, 20 IRSp53_KD-1000, 18 SCR-5000, 18 IRSp53_KD-50000 spheroids from 3 independent experiments). **L-O**. Spreading parameters obtained from image segmentation and PIV analysis of spheroids of different size. L. Quantification of A. M. Quantification of B. N. Nematic length L_c. Data are means ± SD from 3 independent experiments (n=19 SCR-250, 21 IRSp53_KD-250, n=21 SCR-1000, 20 IRSp53_KD-1000, n=13 SCR-5000, 17 IRSp53_KD-5000 spheroids). O. Rate of core melting d(R1/R0)/dt estimated for SCR-5000 and IRSp53-KD-5000 spheroids. Data are means ± SD from 3 independent experiments (n=15 SCR-5000, 12 IRSp53_KD-5000 spheroids). The rate could not be estimated for smaller spheroids as R_1 drops too fast for a clear decrease to be distinguished from the noise.

Statistical analysis for each experiment is included in the Methods section. P values are indicated in each graph.

Supplementary Figure S2

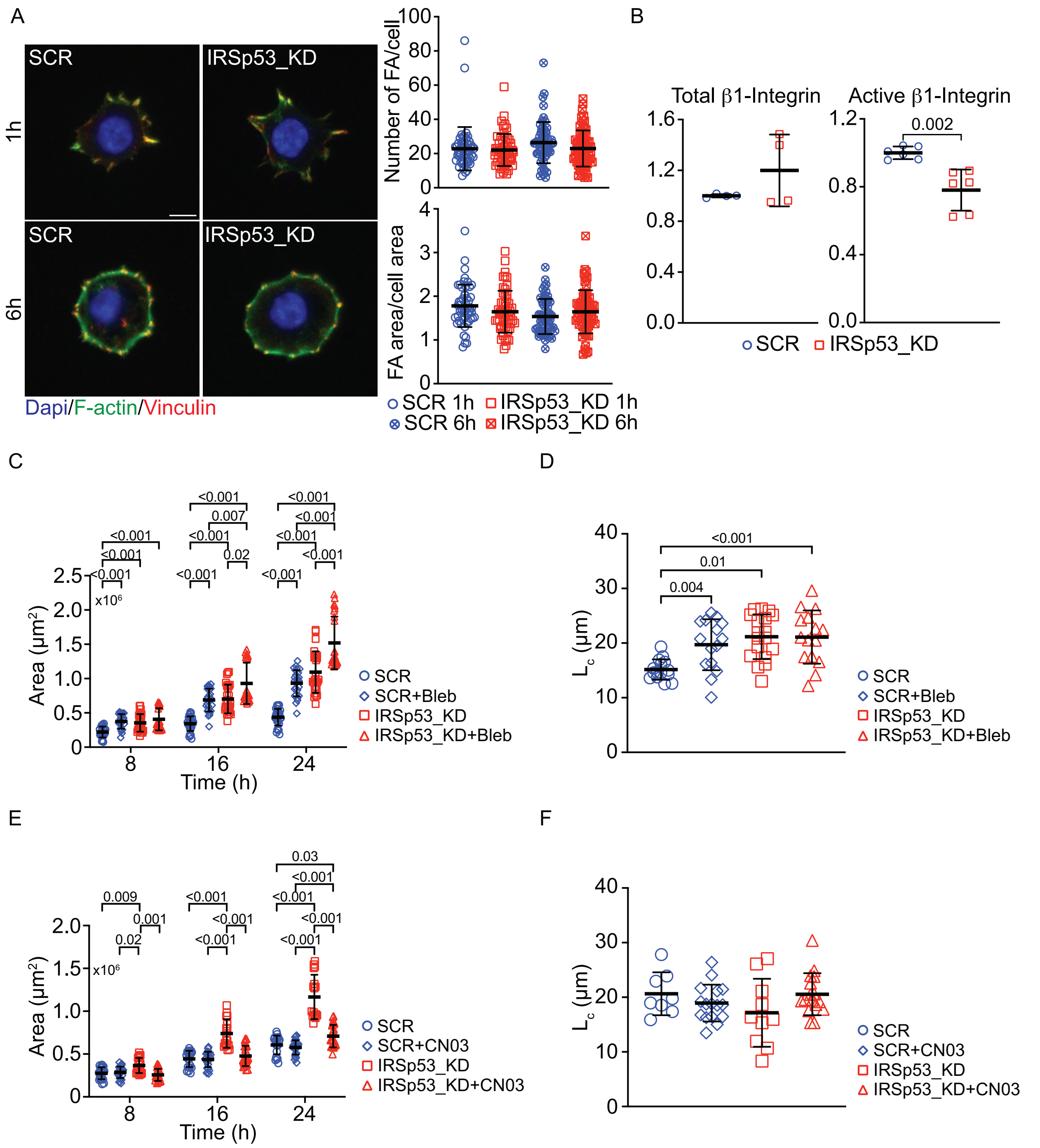

Supplementary Figure S2. **A**. IRSp53 loss does not perturb focal adhesions. Doxycycline-treated MCF10 DCIS.COM control (SCR) or IRSp53 silenced (IRSp53_KD) cells were seeded on fibronectin (10μg/ml) coated glass bottom dishes and fixed 1h or 6h after seeding. Cells were stained with anti-Vinculin antibody (red), FITC-phalloidin to detect F-actin (green), and DAPI (blue) and visualized by TIRF microscopy. Left, representative TIRF images at 1h and 6h are shown. Scale bar, 10μm. Right graphs, quantification of the number of focal adhesions/cell (up) and focal adhesion area/cell area (low) was performed using ImageJ (for details please see Material and Methods section). Data are mean ± SD (1h: n=53 (1210) SCR, 56 (1235) IRSp53_KD cells (focal adhesions) from 2 independent experiments; 6h: n=58 (1546) SCR, 66 (1515) IRSp53_KD cells (focal adhesions) from 2 independent experiments). **B**. IRSp53 removal does not increase β1-integrin surface level. FACS analyses of cell surface total β1-integrin (left) and active β1-integrin (right) in control (SCR) and IRSp53-silenced (IRSp53_KD) MCF10 DCIS.COM cells. Data are represented as mean fluorescence intensity, fraction of SCR ± SD (n=4 technical replicates from n=2 independent experiments for total β1-integrin; n=6 technical replicates from n=3 independent experiments for active β1-integrin). **C**. The spreading area of wetting spheroids (Fig. 4D and Supplementary Movie S11) at the indicated time points was manually quantified using ImageJ. Data are mean ± SD (n=29 SCR CTR, 26 SCR+Blebb, 27 IRSp53_KD CTR, 24 IRSp53_KD+Blebb spheroids from 3 independent experiments). **D.** Quantification of the nematic lengh L_c obtained from image segmentation and PIV analysis of MCF10 DCIS.COM spheroids upon Blebbistatin treatment (Fig. 4D and Supplementary Movie S11). Data are mean ±SD (n=15 SCR, n=15 SCR+Blebb, n=18 IRSp53_KD, n=15 IRSp53_KD+Blebb cells from 3 independent experiments) **E**. The spreading area of wetting spheroids (Fig. 4E and Supplementary Movie S12) at the indicated time points was manually quantified using ImageJ. Data are mean ± SD (n=21 SCR CTR, 26 SCR+CN03, 17 IRSp53_KD CTR, 24 IRSp53_KD+CN03 spheroids from 3 independent experiments). **F**. Quantification of the nematic length L_c obtained from image segmentation and PIV analysis of MCF10 DCIS.COM spheroids upon CN03 treatment (Fig. 4E and Supplementary Movie S12). Data are mean ±SD (n=8 SCR, n=16 SCR+CN03, n=10 IRSp53_KD, n=16 IRSp53_KD+CN03 cells from 3 independent experiments).

Statistical analysis for each experiment is included in the Methods section. P values are indicated in each graph.

Supplementary Figure S3

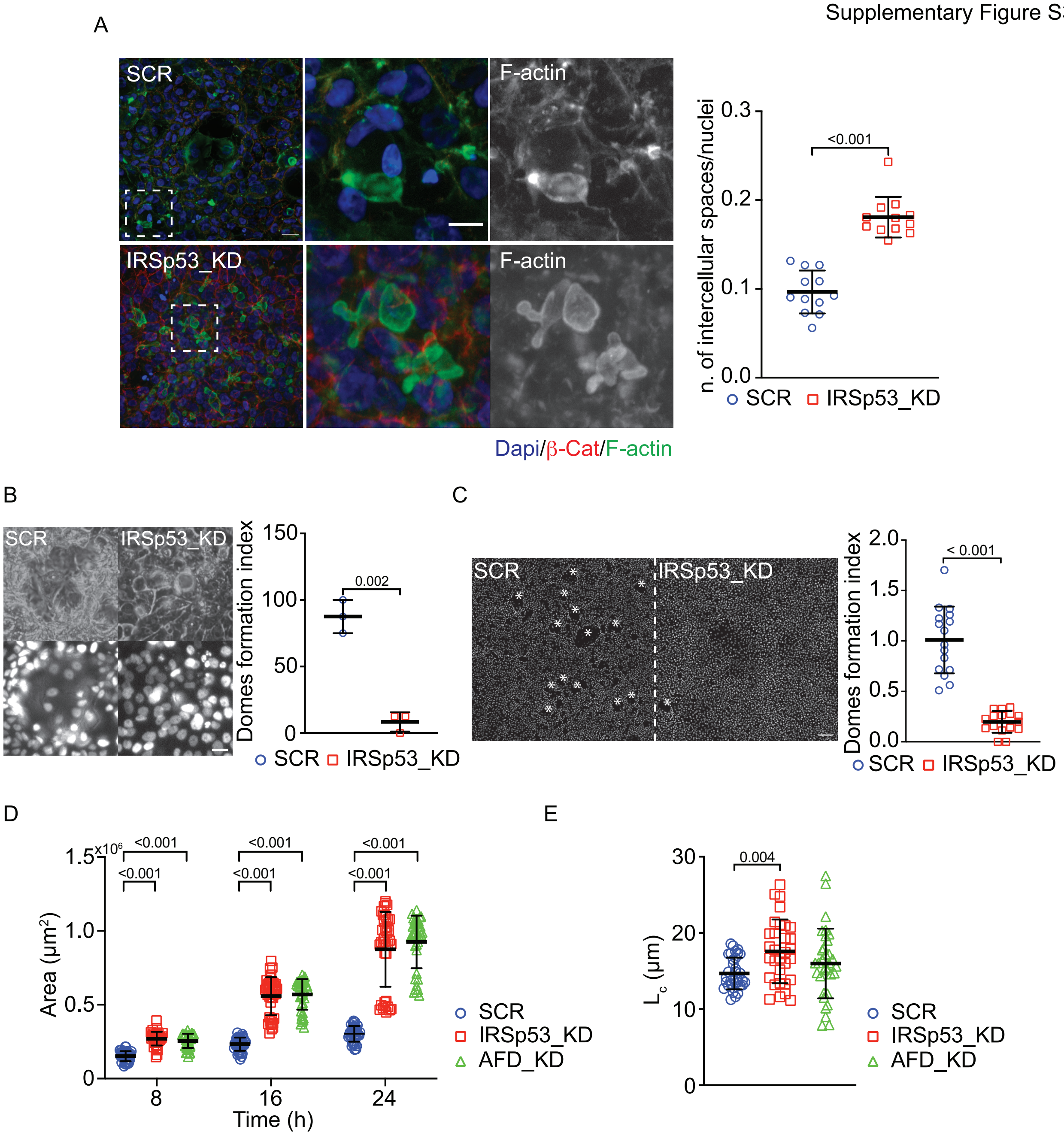

Supplementary Figure S3. **A**. IRSp53 loss increase intercellular spaces formation. Doxycycline-treated SCR and IRSp53_KD MCF10 DCIS.COM cells were seeded at jamming density and fixed 96 hours after seeding. Cells were stained with anti-α-Catenin antibody (Red), FITC-phalloidin to detect F-actin (green), and DAPI (blue). Representative deconvoluted 3D images, acquired with Leica THUNDER imaging system are shown. Scale bar, 10μm. (See also Supplementary Movies S17-18). Right graph, intercellular spaces quantification. The number of intercellular spaces/nuclei was counted. Data are mean ± SD (n=12 fields from 2 independent experiments; 230 nuclei/field SCR, 247 nuclei/field IRSp53_KD). **B**. Doxycycline-treated SCR-H2B-GFP and IRSp53_KD-H2B-mCherry MCF10 DCIS.COM cells seeded at jamming density and monitored by time-lapse microscopy (Supplementary Movies S19). The formation of epithelial domes was observed and counted between 72 and 96h after seeding (Supplementary Movies S19-20). Domes formation index is the number of fields showing domes, over time. Data are mean ± SD (n=24 fields from 3 independent experiments). **C**. Doxycycline-treated SCR and IRSp53_KD (SCR GFP-E-Cad and IRSp53_KD GFP-E-Cad) MCF10 DCIS.COM cells seeded at jamming density and fixed 96h after seeding. Cells were stained with anti-β-Catenin antibody (not shown), TRITC-Phalloidin to detect F-actin and DAPI (or processed for epifluorescence to visualize GFP-E-Cad (not shown) and stained with TRITC-Phalloidin to visualize F-actin (not shown) and DAPI). The presence of holes within the epithelial layers was used as a proxy of domes formation. Domes formation index is the number of domes/fields, normalized to the SCR mean. Data are mean ± SD (n=28 fields from 2 independent experiments). **D**. The spreading area of wetting spheroids (Fig. 7A and Supplementary Movie S21) at the indicated time points was manually quantified using ImageJ. Data are mean ± SD (n=38 SCR, 39 IRSp53_KD, 38 AFD_KD spheroids from 3 independent experiments). **E**. Quantification of the nematic length L_c obtained from image segmentation and PIV analysis of wetting spheroids (Fig. 7A and Supplementary Movie S21). Data are mean ± SD (n=34 SCR, 31 IRSp53_KD, 33 AFD_KD spheroids from 3 independent experiments)

Statistical analysis for each experiment is included in the Methods section. P values are indicated in each graph.

Supplementary Movie_S1. Doxycycline-treated control (SCR), IRSp53_KD and IRSp53_KD stable expressing murine IRSp53 WT (IRSp53_KD+mIRSp53) MCF10 DCIS.COM cells confluent monolayers were scratched to induce directed cell migration during wound healing. Cells were monitored by phase contrast time-lapse microscopy over a 24h period (example trajectories in the first 10h are shown). Samples were analysed with ImageJ software. Time frame 10 min. Scale bar, 100µm.

Supplementary Movie_S2. Doxycycline-treated control (SCR) and IRSp53_KD cells seeded on fibronectin (10µg/ml) coated linear patterns (10µm width). Cells were monitored by phase contrast time-lapse microscopy over a 24h period. Movies were analysed with a developed C++ software coupled with R [55]. Time frame 10min. Scale bar, 100µm.

Supplementary Movie_S3. Doxycycline-treated control (SCR), IRSp53_KD and IRSp53_KD stable expressing murine IRSp53 WT (IRSp53_KD+mIRSp53) MCF10 DCIS.COM cells confluent monolayers were scratched to induce directed cell migration during wound healing. Cells were monitored by phase contrast time-lapse microscopy over a 24h period (first 10h are shown). Time frame 10min. Scale bar, 100µm.

Supplementary Movie S4. Time-lapse phase contrast (top panels) and fluorescence (lower panel) microscopy of SCR or IRSp53_KD cells confluent monolayers, expressing H2B-GFP and H2B-mCherry respectively, approaching a jamming transition phase. Unjamming-to-Jamming transition was monitored over a 72h period (first 48h are shown). Time frame, 10min. Scale bar, 50µm.

Supplementary Movie S5. Collective locomotion of doxycycline-treated empty vector (EV) and RAB5A MCF10 DCIS.COM cells, silenced with oligos for Luciferase (EV_Luc, RAB5_Luc) or human IRSp53 (EV_IRSp53_RNAi, RAB5_IRSp53_RNAi), seeded at jamming density was monitored by time-lapse phase contrast microscopy over a 24h period. Time frame, 5min. Scale bar, 100µm.

Supplementary Movie S6. Time-lapse phase contrast microscopy of control (SCR) or IRSp53_KD MCF10 DCIS.COM spheroids seeded on fibronectin. Pictures were taken every 10 min over a 48h period (first 24h are shown). Scale bar, 20µm.

Supplementary Movie S7. Time-lapse phase contrast microscopy of wild type (WT) or IRSp53_KO murine primary mammary epithelial cells (MECs) spheroids seeded on fibronectin. Pictures were taken every 10 min over a 48h period (first 24h are shown). Scale bar, 100µm.

Supplementary Movie S8. Time-lapse phase contrast microscopy of control (SCR) or IRSp53_KD HaCat spheroids seeded on fibronectin. Pictures were taken every 10 min over a 72h period (first 48h are shown). Scale bar, 100µm.

Supplementary Movie_S9. Time lapse phase contrast (upper panels) and corresponding PIV analysis (lower panels) of doxycycline-treated control (SCR) and IRSp53_KD MCF10 DCIS.COM spheroids seeded on fluorescent beads embedded-, collagen coated-PDMS (15 kPa). Pictures were taken every 10 min over a 15h period. Scale bar, 10µm.

Supplementary Movie S10. Time lapse phase contrast analysis of doxycycline-treated control (SCR) and IRSp53_KD MCF10 DCIS.COM spheroids flowed into microfluidic device design for repetitive deformations (Ref). Pictures were taken every 5ms over ±10s. Scale bar, 100µm.

Supplementary Movie S11. Time-lapse phase contrast microscopy of doxycycline-treated control (SCR) or IRSp53_KD MCF10 DCIS.COM spheroids, treated with DMSO (left panels) or 5µM blebbistatin, seeded on fibronectin (10µg/ml). Pictures were taken every 10 min over a 48h period (first 24h are shown). Scale bar, 100µm.

Supplementary Movie S12. Time-lapse phase contrast microscopy of doxycycline-treated control (SCR) or IRSp53_KD MCF10 DCIS.COM spheroids, treated with vehicle or with 10µg/ml CN03, seeded on fibronectin (10µg/ml). Pictures were taken every 10 min over a 48h period (first 24h are shown). Scale bar, 100µm.

Supplementary Movie S13. Time-lapse microscopy of doxycycline-treated SCR and IRSp53_KD MCF10 DCIS.COM detached cells 5 min after treatment with trypsin and gentle pipetting. Pictures were taken every 10 sec over 10min. Scale bars, 10µm.

Supplementary Movies S14. Laser ablation of cell-cell junctions in SCR and IRSp53_KD MCF10 DCIS.COM cells monolayers stably expressing GFP-CaaX. Pictures were taken every 5 sec. Scale bar, 10 µm.

Supplementary Movie S15. FIB-SEM (focused ion beam-scanning electron microscope) of MCF10 DCIS.COM control (SCR) spheroid. 1770 slices (1495 used) slices were acquired in 45h 25min using a lateral pixel size of 5 nm and the FIB slice thickness of 10 nm. 1495 slices were used of 1770 acquired slices. The ROI imaged was 2560x3150 pixel resulting in a volume of 12,75x15.75x14,95μm^3.

Supplementary Movie S16. FIB-SEM (focused ion beam-scanning electron microscope) of MCF10 DCIS.COM IRSp53_KD spheroid. 2064 slices (1839 used) were acquired in 51 h and 13 min using a lateral pixel size of 2.5 nm and a FIB slice thickness of 5 nm. The imaged ROI of 2100x5550 pixel was resulting in an acquired volume of 5.25x8.33x 9.2μm^3.

Supplementary Movie S17. Intercellular spaces of control (SCR) or IRSp53_KD MCF10 DCIS.COM confluent monolayers. Samples were fixed, processed for epifluorescence and stained as indicated. Z-stack acquisition (step size 0.2µm) was performed using Leica SP8-DLS microscope and images were generated using ImageJ software. Scale bars, 10µm.

Supplementary Movie S18. 3D reconstruction (ImageJ software) of the intercellular spaces described in Supplementary Movie S13.

Supplementary Movie S19. Cropped images of control (SCR) or IRSp53_KD MCF10 DCIS.COM cells confluent monolayers stably expressing H2B-GFP or H2B-mCherry respectively. Collective locomotion was monitored by time-lapse phase contrast (top panels) and fluorescence (lower panels) microscopy. Time frame, 10min. Scale bar, 50µm.

Supplementary Movie S20.
3D reconstruction (ImageJ software) of a dome generated by control (SCR) DCIS.COM cells super-confluent monolayer. Sample was fixed and stained as indicated for microscopy analysis. Z-stack acquisition (step size 0.2μm) was performed using Leica Thunder microscope.

Supplementary Movie S21. Time-lapse microscopy of doxycycline-treated MCF10 DCIS.COM control (SCR), IRSp53 silenced (IRSp53_KD) or Afadin silenced (AFD_KD) spheroids seeded on fibronectin (10μg/ml). Images were taken every 10 min over a period of 48h period (first 24h are shown). Scale bar, 200μm.

Supplementary Movie S22. Laser ablation of apical area of doxycycline-treated MCF10 DCIS.COM control (SCR), Afadin silenced (AFD_KD) or IRSp53 silenced (IRSp53_KD) cells monolayers stably expressing mCherry-CaaX. Pictures were taken every 3 sec. Scale bar, 10μm.

References

55 Maiuri, P. *et al.* The first World Cell Race. *Curr Biol* **22**, R673-675, doi:10.1016/j.cub.2012.07.052 (2012).
